# Light-controlled soft bio-microrobot

**DOI:** 10.1038/s41377-024-01405-5

**Published:** 2024-02-26

**Authors:** Jianyun Xiong, Xing Li, Ziyi He, Yang Shi, Ting Pan, Guoshuai Zhu, Dengyun Lu, Hongbao Xin

**Affiliations:** Guangdong Provincial Key Laboratory of Nanophotonic Manipulation, Institute of Nanophotonics, Jinan University, 511443 Guangzhou, China

**Keywords:** Biophotonics, Biomedical materials, Optical manipulation and tweezers

## Abstract

Micro/nanorobots hold exciting prospects for biomedical and even clinical applications due to their small size and high controllability. However, it is still a big challenge to maneuver micro/nanorobots into narrow spaces with high deformability and adaptability to perform complicated biomedical tasks. Here, we report a light-controlled soft bio-microrobots (called “Ebot”) based on *Euglena gracilis* that are capable of performing multiple tasks in narrow microenvironments including intestinal mucosa with high controllability, deformability and adaptability. The motion of the Ebot can be precisely navigated via light-controlled polygonal flagellum beating. Moreover, the Ebot shows highly controlled deformability with different light illumination duration, which allows it to pass through narrow and curved microchannels with high adaptability. With these features, Ebots are able to execute multiple tasks, such as targeted drug delivery, selective removal of diseased cells in intestinal mucosa, as well as photodynamic therapy. This light-controlled Ebot provides a new bio-microrobotic tool, with many new possibilities for biomedical task execution in narrow and complicated spaces where conventional tools are difficult to access due to the lack of deformability and bio-adaptability.

## Abstract (web summary)

We report a light-controlled soft bio-microrobots (called “Ebot”) based on *Euglena gracilis* that are capable of performing multiple tasks in narrow and changeable microenvironments with high controllability, deformability and adaptability.

## Graphical abstract

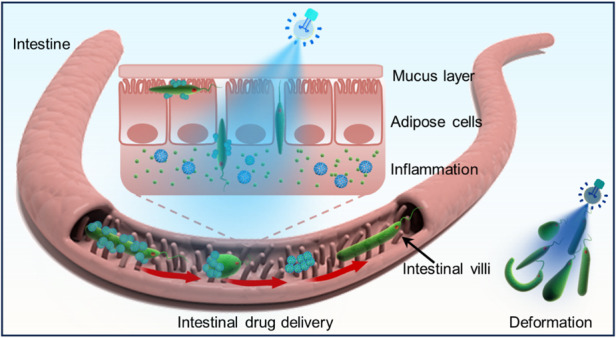

## Introduction

Micro/nanorobots have attracted much attention in the fields of biomedicine and biomedical engineering due to their smaller size compared with traditional tools, and they can be precisely navigated to hard-to-reach areas of bio-microenvironments and even the human body to perform medical tasks^1^. Due to the controllable motion and good biocompatibility, micro/nanorobots are widely used in many biomedical applications, such as targeted drug delivery, thrombus removal, precise therapy and microsurgery^2–7^. Although conventional rigid micro/nanorobots have attractive potentials in biomedical applications, they are still very difficult to execute complicated tasks on soft and fragile bio-targets, particularly in complicated and narrow spaces where the robots are difficult to access or pass through (such as blood vessels and intestines)^8^. Therefore, it is very important to design a soft micro/nanorobot that can pass through such narrow environments and can change the shape to adapt to the microenvironments to perform different tasks.

Compared with traditional rigid microrobots, soft microrobots with deformability and adaptability properties are particularly promising to perform different tasks in complicated environments and sinuous microchannels to satisfy the requirements of biomedical applications^9–11^. Nevertheless, there are many technical challenges in designing and fabricating soft microrobots with high biocompatibility. Fortunately, nature can always teach us and inspire us^12^. In nature, many soft organisms can adapt to different environmental changes by changing their shapes and motion directions. For example, octopus with a soft body exhibits extremely high deformability and flexibility^13,14^, and it can squeeze its shape to pass through narrow holes to capture different targets^15^. Caterpillar can curl its slender body into a wheel that can roll and flee from danger^16^. Earthworm can change their shape to quickly pass through winding and narrow pipes to avoid damage^17^. Due to the evolution of these soft-bodied organisms over millions of years, these organisms exhibit multifunctional locomotion patterns with strong adaptability to hostile environments, which greatly increases the possibility for survival and reproduction. Inspired by these soft organisms, researchers have designed soft microrobots through various actuation mechanisms^18–22^. For example, octopus-inspired and magnetically controlled soft microrobots composed of biocompatible hydrogels have been designed for targeted drug delivery in vivo^23^. However, for magnetic-driven methods, modification of magnetic materials is needed. In addition, the bulky size of the magnetic field generation device also limits microrobotic integration. Alternatively, light-controlled mechanism has become another flexible way to drive bioinspired soft microrobots with high spatiotemporal resolution^24,25^. For example, a soft microrobot driven by structured light was developed that can wriggle and self-propel through periodic body deformation without external force^26^. However, in order to facilitate the fabrication of bioinspired soft robots, their structures are often greatly simplified. As a result, the task-perform capability of these bioinspired soft robots cannot match that of their natural counterparts^27^. To solve this problem, researchers find new solutions and directly use microorganisms to construct bio-hybrid soft microrobots with multifunctional capabilities^28–32^. For example, microalgae are widely used in biomedical research such as drug loading and delivery^33–36^. However, the reported *Chlamydomonas reinhardtii* (CR) microalgae is not soft organism. Besides, it should be noted that at the microscopic scale, the movement of organisms in liquids is fundamentally different from swimming at the macroscopic scale^37,38^. In particular, due to the relatively large viscous and frictional forces (usually several orders of magnitude higher than the gravity)^39^, it is very difficult to effectively control the movement and deformation of soft microorganisms to construct soft bio-microrobots to perform various complex tasks.

**Fig. 1:**
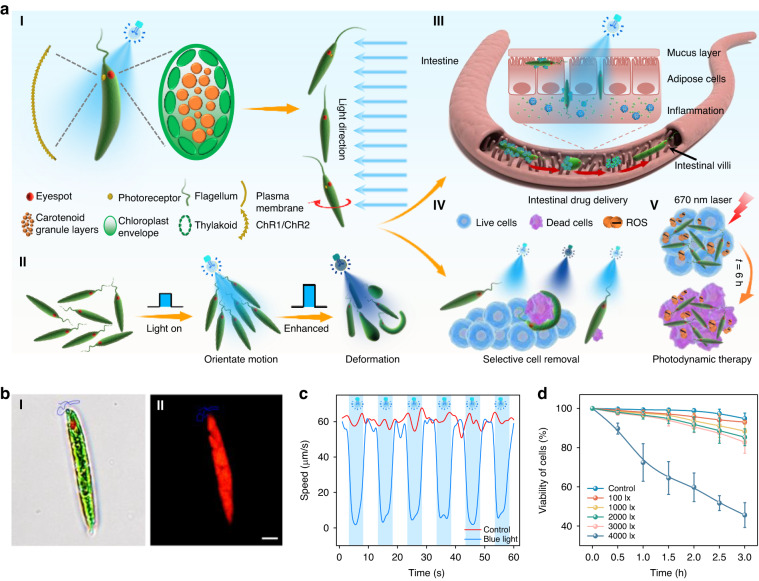
Characterization of EG cells and Ebots. **a** Schematic illustration of EG structure and multifunctional applications of Ebots. (I) Eyespot structure and phototaxis behavior of EG. (II) Phototaxis and deformation of EG cell. (III-V) Schematic illustration of Ebot for (III) targeted drug delivery, (IV) selective removal of diseased cells in intestinal mucosa and (V) PDT. **b** (I) Phase contrast microscopy and (II) fluorescent microscopy images of EG. **c** Swimming speeds of Ebot in the natural state and under periodic light irradiation, respectively. **d** The viability of Ebot as a function of time during light irradiation of different light intensities. Scale bar: 5 μm. Data for (**d**) are presented as mean values ± s.d. (*n* = 18)

In this work, we report light-controlled multifunctional soft bio-microrobots (called “Ebot”) directly using microalga *Euglena gracilis* (EG). Such Ebot exhibits strong deformability and adaptability which ensures the task-execution capability in narrow bio-microenvironments including intestinal mucosa controlled by blue light (Fig. 1a). The motion of the Ebot can be precisely controlled by the multimodal flagellum swing controlled with different light intensity, while the shape can be controlled with different light irradiation duration. With the feature of deformability and adaptability in different environments, Ebot is able to execute multiple tasks, such as targeted drug delivery, selective removal of apoptotic cells in the intestinal mucosa, as well as photodynamic therapy (PDT).

## Results

### Characterization of Ebot

Natural EG (average length *l*: 50 μm, width *w*: 9 μm, Fig. 1b-I and Fig. S1) exhibits good motility in natural environment in a rotary-forward mode. EG exhibits red fluorescence upon 540 nm green light excitation due to a large amount of chlorophyll (Fig. 1b-II). Without external light irradiation, the motion of EG is usually in a random mode (Fig. S2a). However, EG is a photosensitive euglena with photoreceptor at the back of the eyespot, and is especially sensitive to blue light (wavelength 400–500 nm)^40^. To show the effective response of EG to different light irradiation, we carried out experiments using sunlight, UV light (wavelength: 350 nm), blue light (wavelength: 450 nm), green light (wavelength: 540 nm), and red light (wavelength: 670 nm). As shown in Fig. S3, we found that the movement pattern of Ebot was not changed under the irradiation of green light and red light. Instead, the motion is changed by sunlight, UV, and blue light. But the motion response is much higher for blue light than that of sunlight and UV. This is because blue light can activate adenylate cyclase in photoreceptors, which can phosphorylate proteins in flagellum, resulting in changing of flagellum beating patterns^40^. Therefore, blue light was used to control Ebot’s movement in our work. Here, we chose a blue light-emitting diode (LED) with a center wavelength of 450 nm as the light source to control the EG^41^. Upon light irradiation, EG responds to light and swims stably toward the light source, exhibiting phototaxis (Fig. S2b). Photoreceptor and eyespot are crucial for the phototaxis behavior of EG. As shown in Fig. 1a-I, the eyespot is composed of irregularly arranged carotenoid particles of different sizes (240 ~ 1200 nm in diameter). Such structure results in a transmittance of only 30% ~ 40% for the eyespot, and the eyespot is therefore also regarded as a shading device^42,43^. The plasma membrane on photoreceptors is rich in channelrhodopsin-1 (ChR1) and channelrhodopsin-2 (ChR2) proteins^44^, which can absorb light energy and transmit it to flagellum. The energy transmitted to flagellum can change the flagellum swing mode, which eventually results in the change of EG motion direction. During the motion upon light irradiation, the rolling motion of the EG makes the eyespot block the photoreceptors from receiving light signals at a frequency of 1–2 Hz. On one hand, the amount of energy transmitted to flagellum can change the swing mode of flagellum^45^, which can eventually change the motion mode of the EG. So the EG needs to dynamically adjust its motion direction to maintain continuous reception of optical signals. Therefore, the motion direction of the EG can be controlled and navigated by light. On the other hand, EG can deform and change its shape to adjust to a new environment with changing conditions^46^. In our case, when the light intensity is 3000 lx, the increased photon energy received by photoreceptors can trigger the EG flagellum malfunction and causes the EG to deform (Fig. 1a-II). Combining these two effects, the EG can then be turned into a living soft Ebot controlled by light. This EG can execute biomedical tasks, such as drug delivery and selective removal of diseased cells in narrow and winding microenvironment such as intestinal mucosa (Fig. 1a-III and Fig. 1a-IV), as well as PDT at targeted locations (Fig. 1a-V). To show the light response and motion controllability of EG, periodical irradiation of EG was carried out. As shown in Fig. 1c, without light irradiation, the EG randomly moves at an average speed of 60 μm/s. Upon light irradiation, as a response to the light irradiation (light energy absorption by photoreceptors and energy transition to flagellum), the Ebot stops in the original position for 0.5 ~ 1 s and then reselects the subsequent motion direction for further movement. It should be noted that, for light intensity lower than 3000 lx, no significant affects are observed on the viability and activity of EG cells with light irradiation up to 3 h (Fig. 1d). When the light intensity reached 4000 lx, the Ebot activity decreased significantly. Therefore, in our following experiments, to avoid photodamage to the EGs, light irradiation with intensities 3000 lx was used to command the Ebot to move according to the specified instructions to execute different tasks.

### Motion control of Ebot

**Fig. 2:**
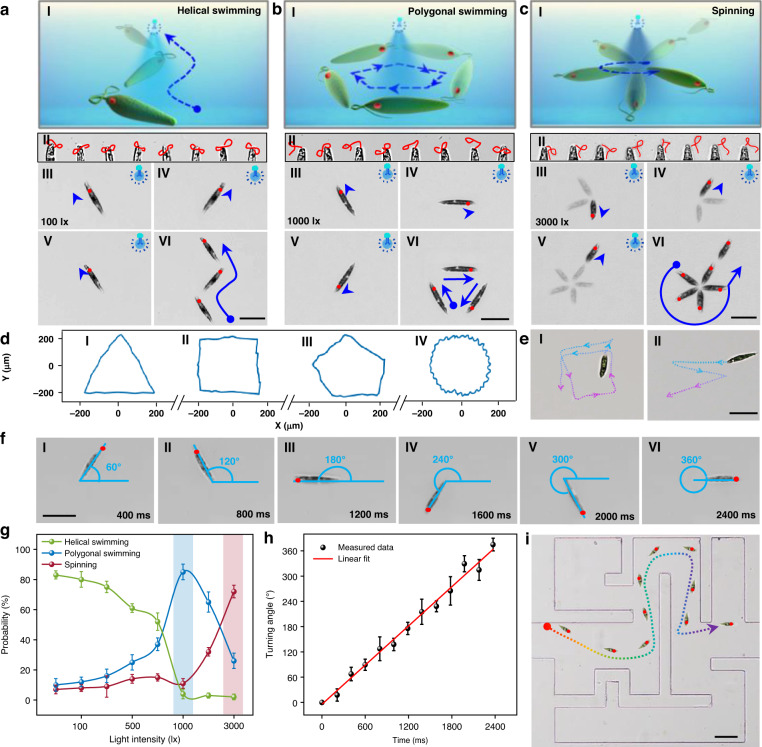
Motion control of the Ebot. **a**–**c** Three different motion modes of (**a**) helical swimming under low intensity light, (**b**) polygonal swimming under medium intensity light, and (**c**) spinning swimming under high intensity light. Panels I: schematic illustration of these three motion modes, blue curves show the motion strategy. Panels II: microscopic images showing the flagellum beating patterns for each motion mode, red curve indicates the flagellum. Panels III-V: microscopic images showing the movement of the Ebot. Red dot indicates the eyespot position. Panels IV: microscopic images showing final swimming trajectories with several images superimposed (blue curves indicated). **d** Different polygonal trajectories of Ebot at medium light intensity. **e** Microscopic images showing different directions of polygonal trajectories. **f** Microscopic images showing the effect of illumination duration on the turning angle of the Ebot under high intensity light. **g** Probability of different motion modes as a function of light intensity. **h** Measured turning angle under different illumination duration. **i** Microscopic image showing Ebot passing through a microfluidic maze, dashed curve indicates the Ebot trajectory. Scale bars: 50 μm. Data for (**g**) and (**h**) are presented as mean values ± s.d. (*n* = 20)

The controllable motion and reorientation of EG under light irradiation are crucial for Ebots to execute different biomedical tasks. To analyze the motion control capability of blue light on Ebot, we explored the motion mode of EG under blue light irradiation with different light intensities (Fig. 2g). We find that, there are three typical motion modes of the Ebot, which are helical swimming, polygonal swimming, and spinning (Fig. 2a–c, Movie S1). This is resulted from the different beating patterns of flagellum as EG adapts to different light illumination with different intensities.

As shown in Fig. S4, the deformation and rotation of EG are strongly related to the photoreceptors, eyespots and flagellum in the head position of EG (Fig. S4-I). Among them, the photoreceptor is the device that receives light, the eyespot serves as a light-shielding device, and the flagellum with different swing modes controls the movement direction and deformation of the EG. Therefore, the swing of the flagellum is the key factor in motion control of EG. According to Porter et al., the reason for flagellum showing different swing modes is related to their “9 + 2” axonemal structure^47^, which includes 9 double-linked microtubules near the outer boundary and 2 single-linked microtubules in the center (central microtubule) (Fig. S4-II). When the photoreceptor receives light, it can stimulate Channelrhodopsin-2 (ChR2) on the flagellar membrane that controls the exchange of Ca^2+^, and the Ca^2+^ concentration level inside the flagellum is increased (Fig. S4-III). The Ca^2+^ concentration of the flagellum will affect the rotation of the central microtubule, which will further affect the rotation of outer double-linked microtubules through the connecting radial spoke. This rotation finally results in the different swing modes of the flagellum. With a weak-intensity light illumination (for example, 100 lx), the ChR2 channel is not activated, and the Ca^2+^ concentration is not changed; thus the flagellum maintains the natural “figure eight” mode, and the EG swims along a helical path (Fig. S4-IV, Fig. 2a). With a medium-intensity light illumination (1000 lx), the ChR2 channel can be activated, and the Ca^2+^ concentration is increased. The swing mode of the flagellum is changed to a “rotating lasso”, and the EG turns sideways. After the turning, the light is blocked by the shading of the eyespot, and the Ca^2+^ concentration recovers to the normal state. The EG thus swims in the natural helical state with flagellum swinging in “figure eight” pattern until light is detected by the photoreceptor again. This process will repeat periodically, and the motion is eventually in a polygonal trajectory (Fig. 2b). However, for cells at different times, due to the variation in cellular activity and metabolism, the turning angle can be different when sensing the light illumination, and thus the polygonal trajectories can be different (Fig. 2d, Movie S2). In addition, the direction of the flagellum beating in the “rotation lasso” can also be different after the turning, and thus the polygonal trajectories can also be in different directions (Fig. 2e). Therefore, for the polygonal motion under medium-intensity light irradiation, it is not easy to precisely control the motion behavior of the Ebot.

With light irradiation at a high intensity (3000 lx), once the EG senses this strong light stimulus, it turns sideways. After this turning, although part of the light is blocked by the eyespot, the photoreceptor can still sense the transmitted light from the eyespot. In this case, the ChR2 channel is always at an activation state, and the Ca^2+^ concentration is also at an increased state. Therefore, the flagellum is at the “rotating lasso” mode of swing, which eventually results in the local spinning of the Ebot (Fig. 2c). By turning off the light illumination, the Ebot recovers the natural helical swimming. Importantly, the turning angle during the spinning can be controlled with different illumination durations. As shown in Fig. 2f, when the illumination time was 400 ms, the Ebot rotated 60° and then started the natural helical swimming. The turning angle was increased to 120°, 180°, 240°, 300°, and 360° as the illumination time was increased to 800, 1200, 1600, 2000, and 2400 ms, respectively. Further experimental results show that the turning angle of the Ebot is linearly increased with increase illumination time (Fig. 2h, Movie S3). These results demonstrate that under high-intensity light illumination, the motion and navigation direction of the Ebot can be controlled. Specifically, as shown in Fig. S5, under the light illumination of 3000 lx, the Ebot can be controlled to a designated orientation angle (30°) with a given illumination duration (200 ms), and then the Ebot can swim forward helically to the next stop by turning off the illumination light. This process then repeats and the Ebot can be navigated to the final targeted position for further applications. Based on this strategy, as shown in Fig. 2i (Movie S4), we successfully controlled the Ebot to pass through a microfluidic maze (see Method for fabrication details). Without light control, the Ebot was lost in the maze (Fig. S6). In the process of motion control of the Ebot, the whole field of view is illuminated by the LED light. In addition to the control of a single Ebot, we can also control multiple Ebots in the field of view simultaneously, which is similar to the control of a single Ebot. As some examples, Fig. S7 shows the simultaneous control of two Ebots, including helically forward swimming, polygonal swimming, and spinning. In addition to the control of EG, we can also control the motion of other phototactic algae CR and *volvox* due to the phototaxis, as shown in Fig. S8. However, the CR and *volvox* show no deformation capability.

### Deformation control of Ebot

**Fig. 3:**
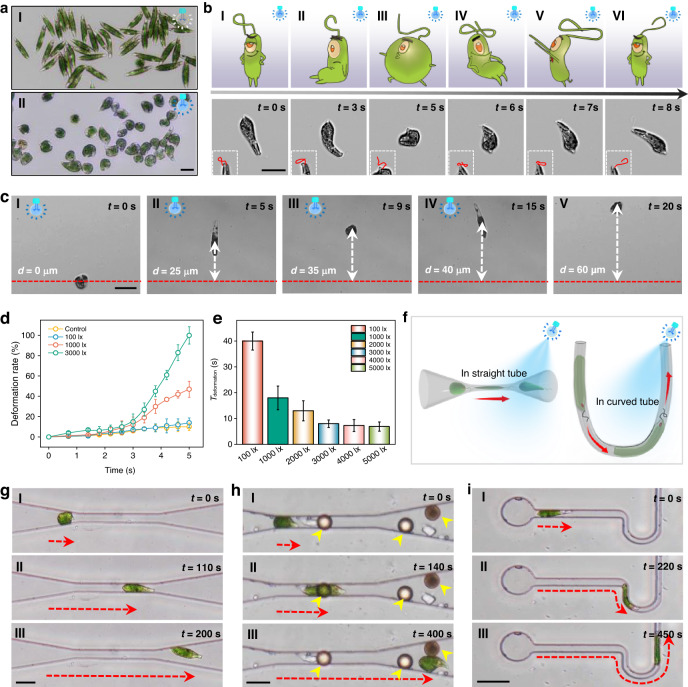
Deformation control of Ebot. **a** Microscopic images showing overall observation of deformation by light irradiation. Panel I: without light irradiation, no deformation is observed. Panel II: under light illumination (3000 lx), Ebot deformation is observed with various shapes. **b** Deformation progress of Ebot, upper panels: schematic illustrations, lower panels: microscopic images. Insets are microscopic images showing beating patterns of malfunctional flagellum. **c** Microscopic images showing Ebot moving forward during light-induced deformation. Red dashed lines indicate the initial position of Ebot, and white dashed arrows indicate the moving distance of Ebot. **d** Deformation rate of Ebot as a function of time under light irradiation with different intensities. **e** Deformation cycle under light irradiation with different intensities. **f** Schematic illustration of Ebot passing through different microfluidic channels. The red arrows indicate the movement direction. **g–i** Microscopic images showing Ebot navigating in (**g**) a 2D straight channel, width: 10 μm, (**h**) a 3D straight channel, width: 10 μm, and depth: 5 μm, (**i**) a curved channel, width: 5 μm. Red dashed arrows indicate the motion direction of the Ebot. The yellow arrows in (**h**) indicate obstacles. Scale bar: 20 μm. Data for (**d**) and (**e**) are presented as mean values ± s.d. (*n* = 20)

In addition to the control of motion and navigation, the deformation capability of the EG as a soft microrobot is also very important to execute different tasks in a narrow environment. The occurrence of the EG deformation is mainly caused by the irregular and rapid swing of the flagellum. Previous study shows that the flagellar function can be disturbed by different physical factors such as surface obstruction, sticking, resistance, and restriction in limited space during normal beating. Such factors can lead to the irregular and rapid swing of the flagellum and ultimately causes EG to exhibit various deformations^48^. In this study, we find that continuous irradiation by high-intensity light can also cause the irregular and rapid swing of the flagellum, and EG exhibits deformations such as bending, stretching and contracting. As the irradiation at high intensity (3000 lx) continues (more than 3 s), the ChR2 channel is continuously at the excited state, and the Ca^2+^ level inside the flagellum is continuously at an increased state. In this case, the central paired microtubule will rotate too fast, and the “rotating lasso” mode of swing is disrupted (Fig. S4-V), resulting in the rapid and irregular swinging of the flagellum^49^. The regular rotation movement of the EG is also disrupted. Since the flagellum is located in the flagellar pocket built by the microtubules, the flagellar pocket is closely connected to the striated cell envelope (pellicle) of the EG epidermis. Therefore, the irregular and rapid swing of the flagellum will drag the microtubules in the flagellum pocket, which in turn will drive the pellicle of the EG epidermis to slide, causing the EG to deform between a spindle to a sphere. In the unconstrained space without light irradiation, EG cells were spindle-shaped (Fig. 3a-I). However, when a high-intensity light (3000 lx) was applied to it for 5 s. As shown in Fig. 3a-II, and EG exhibited body deformations such as contracting. To further verify the effect of the irregular and rapid swing of the flagellum caused by light on EG deformation, as shown in Fig. 3b, light irradiation (3000 lx) with different durations was applied to a targeted EG. At *t* = 3 s, the EG started to deform slightly with a deformation rate (change in aspect ratio of EG) of about 15%. With a light irradiation of 5 s, the shape of the EG was changed from spindle to spherical shape and the deformation rate reached 100%. It is worth noting that the shape of the EG returned to its initial spindle shape as the light source continued to irradiate for 8 s (Movie S5). By observing the flagella beating (insets in Fig. 3b), we find that the flagellum beats irregularly during light irradiation. This suggests that high-intensity light irradiation for more than 3 s can result in irregular flagellum beating, which is effective in controlling EG deformation in an unrestricted space. In this regard, the EG is turned into an Ebot with controllable deformation by light.

Importantly, during the deformation process, the Ebot can be controlled to perform directional movement by wriggling forward with periodic deformation under high-intensity light irradiation. As shown in Fig. 3c, with light irradiated on an immotile Ebot, the Ebot moved steadily forward accompanied by periodic deformation in unconstrained space. At *t* = 20 s, the Ebot moved forward with a distance of 60 µm.

To further demonstrate the effect of light intensity of irradiation on Ebot deformation, we tested the effect of irradiation with different light intensities on Ebot deformation rate. For irradiation 100 lx, no significant deformation of Ebots was observed (Fig. S9). For irradiation with intensity of 3000 lx, significant deformation (15%) was observed after about 3 s, and the deformation rate was nearly linearly increased with time (Fig. 3d). With an irradiation duration of 5 s, a deformation rate of about 45% and 100% was observed for the light intensity of 1000 and 3000 lx, respectively. The deformation period (the time required for Ebot to deform from a spindle shape to a spherical shape and back to a spindle shape) was about 40, 18, and 8 s for the light intensity of 100, 1000, and 3000 lx, respectively (Fig. 3e).

This controllable deformation and directional movement capability of the Ebot makes it possible to pass through different restricted narrow spaces which are otherwise impossible for rigid tools, as schematically shown in Fig. 3f. To show this capability, we performed different experiments of Ebots passing through different restricted microfluidic channels. As some examples, Fig. 3g–i shows the rapid traversing of Ebot through restricted channels due to the deformation and forward movement of Ebot upon light irradiation. As a first example, we show the successful passing through a two-dimensional (2D) restricted space (an uncovered microfluidic channel with a width of 5 μm and length of 100 μm). For the case without light irradiation, although the irregular and rapid swing of the flagellum and deformation can also happen in restricted space^48^, the EG slowly traverse the channel for 400 s (Fig. S10). For the situation with light irradiation (3000 lx), the Ebot quickly passed through the same channel, and the traversal time was decreased to 200 s (Fig. 3g, Movie S6). In addition to passing through 2D restricted channel, the Ebot can also pass through three-dimensional (3D) restricted space. Here, a 3D restricted channel (width: 5 μm, depth: 7 μm) was designed by covering the channel with a 5-μm polystyrene particle as an obstacle. Without the stimulation of light irradiation, the EG shrinked into a spherical shape after encountering the obstacle and turned back to its original position (Fig. S11). Under the stimulation of light irradiation with 3000 lx, the Ebot moved across the obstacle flexibly (Fig. 3h, Movie S7). Importantly, the Ebot can even pass through a curved channel with a width much smaller than Ebot’s size. As shown in Fig. 3i, the Ebot successfully pass through a curved restricted space with a width of only 3 μm within 450 s under light stimulation. In contrast, without light illumination, the EG was unable to pass through the same curved confined channel (Fig. S12, Movie S8). These results show that the EG can be turned into a controlled soft Ebot to pass through complicated confined spaces via light irradiation with the intensity of 3000 lx, providing new possibilities to perform different tasks in such confined spaces and narrow bio-microenvironments. Therefore, in the following experiments, we used the Ebot to execute different multifunctional biomedical tasks with light irradiation under intensity of 3000 lx.

### Multifunctional biomedical task execution

It is of great importance, but meanwhile also very challenging, to flexibly and precisely control micro/nanorobots to execute different tasks in complicated bio-microenvironments. Remarkably, all the materials of our Ebot are EG cell. This feature makes our Ebot highly biocompatible and biodegradable. The viability of HeLa cells was not affected during co-culturing of EG with HeLa cells for 1 day (Fig. S13), and EGs degraded into fragments after incubation in simulated intestinal fluid (SIF) at 37 °C for 72 h (Fig. S14). These biocompatibility and biodegradation features make our Ebot a good choice for further biomedical applications. Here, our light-controlled soft Ebot can be navigated to designated locations to perform specific functional tasks, such as selective removal of diseased cells in cell clusters and targeted drug delivery in the intestinal tract in vitro, as well as PDT.

**Fig. 4:**
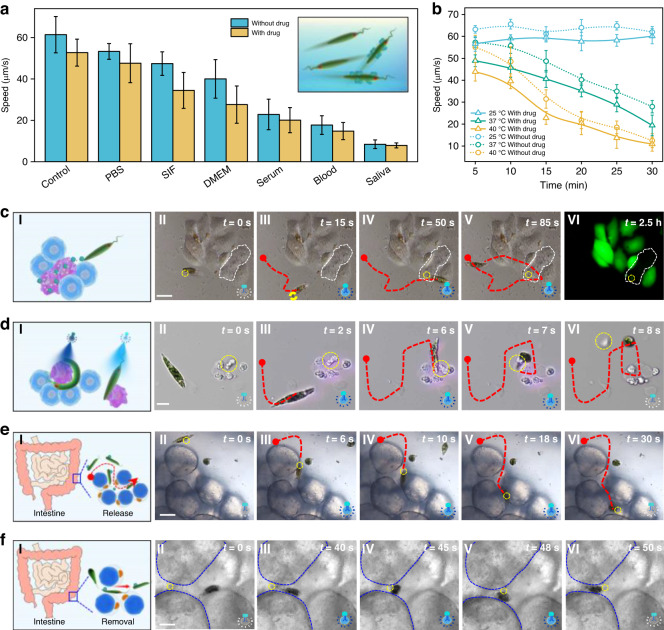
Ebots for multifunctional biomedical task execution. **a** Motion speeds of naked and drug-loaded Ebots in different biological fluids. **b** Speed comparison of naked and drug-loaded Ebots in different temperature. **c–f** Ebot for multifunctional biomedical task execution. Panels I are schematic illustrations, panels II-VI are microscopic images at different time. **c** Targeted drug delivery in cell clusters. Panel VI is fluorescent image, live HeLa cells are green fluorescent. The yellow and white dashed circle indicates the DLSP and cultured HeLa cells, respectively. Red dashed curve shows the trajectory of the Ebot. **d** Selective removal of diseased cells in cell clusters. The yellow dashed circle indicates the target cell. **e** Intestinal-targeted drug delivery in vitro. The yellow dashed circle indicates the DLSP. **f** Removal of porous silica particle in the gut gap in vitro. The yellow and blue dashed circle indicates the particle and the adipocytes in the intestine, respectively. Scale bar: 30 μm. Data for (**a**) and (**b**) are presented as mean values ± s.d. (*n* = 16)

To demonstrate the capability of Ebots for drug delivery, doxorubicin (DOX), a commonly used clinical first-line chemotherapy drug, was loaded into 1-μm mesoporous silica particles. Before the drug delivery performance, we tested the effect of DOX on the viability of the EG cell. Experimental results show that the effect of DOX on the viability of EG is strongly dependent on the concentration of DOX. Direct culturing EG cells in culturing medium mixed with DOX (50 mg/mL) can affect the viability of EG. However, when loaded into the 1 μm porous silica particles, no obvious effect of DOX on the viability of EG was observed (Fig. S15). In order to distinguish the spontaneous red fluorescence of the Ebot, the surface of DOX-loaded silica particles (DLSP) were modified with fluoresceine isothiocyanate (FITC) to make they appear green fluorescence (Fig. S16, see Materials and Methods for details). DLSP were loaded onto the surface of Ebots through electrostatic interactions, and fluorescence microscopy images confirmed the effective combination of DLSPs and Ebots (Fig. S17). For drug delivery, the motion capability in different biological fluids is very important. As shown in Fig. S18, bare Ebots can swim smoothly in different biological media such as EG culture medium, phosphate-buffered saline (PBS), SIF, dulbecco’s modified eagle medium (DMEM), serum, plasma, and saliva. Importantly, drug-loaded Ebot shows similar motion capability, with a slightly smaller velocity than that of bare Ebots (Fig. 4a). In addition, we also tested the motion capability of Ebot at different temperatures, which is important for further biomedical applications such as intestinal drug delivery. Figure 4b shows the motion speed at different temperatures of 25 °C (room temperature), 37 °C (body temperature), and 40 °C. The speed of Ebot was maintained at the initial speed of 60 μm/s within 30 minutes at 25 °C, while the speed was decreased by 50% to only 30 μm/s within 30 minutes at 37 °C. This indicates that a high temperature can affect the motility state of Ebots. Further experiments showed that both pH of the environment and temperature can affect the motion of the Ebot (Fig. S19). To avoid the effects of pH and temperature on the motion Ebot, during the applications, we controlled the pH in a neutral or slightly alkaline state and the temperature at room temperature (25 °C). For applications in other environments with different pH or temperature, we can protect the Ebot by encapsulating Ebot into degradable capsules. It should be noted that microalgae can be used as living dynamic sensor. For example, Roxby et al. reported the sensing of metal ions using a microalgae-based living dynamic sensor^50^. In our case, as shown in Fig. S19a, the motion of our Ebot is sensitive to the pH of the surrounding environment. Therefore, we can take this advantage to make our Ebot a living dynamic pH sensor.

We then showed the drug delivery capability of Ebot for targeted cells among a cluster of HeLa cells. By controlling the deformation of drug-loaded Ebot, DLSP was delivered and released to a targeted cell among the cell clusters with 50 s. After further incubation for 2.5 h, the cell with drug released was dead, while the neighboring cells remained alive (green fluorescence) (Fig. 4c). This result show that the targeted drug delivery capability of the Ebot allows the selective killing of target cells, without affecting the activity of surrounding cells. Although direct shining of UV light can be used for cancer cell killing, the lack of selectivity will affect all cells (both cancer cells and healthy cells). The drug delivery of our Ebot can be used for the killing of one or a few designated cells among a cell cluster with high precision and selectivity without affecting the activity of neighboring cells. This is very important when we need to selectively kill some target cells with high precision. More importantly, when using Ebot as a carrier for drug delivery, particles with different sizes from the nanoscale to the microscale can be loaded or moved. For example, Fig. S20a shows the delivery of 200 nm polystyrene (PS) particles, which cannot be directly moved by optical tweezers due to the diffraction limit, while Fig. S20b shows the delivery of a particle cluster with a size larger than 100 μm, which is even larger than the Ebot itself. The drug/particle loading/collection of the Ebot is realized by electrostatic interaction between the negatively charged EG surface and lysine-modified drug/particles (positively charged), which generally takes about 3 h. The triggering of drug/particle release from the Ebot is realized by the controllable Ebot deformation. After five cycles (about 45 seconds) of Ebot deformation between spindle to spherical shape, the loaded drug/particles can be completely released from the Ebot. Such drug/particle loading and release is repeatable. As shown in Fig. S21, the loading and release of (PS) particles with different sizes of 0.2, 3, 5, 20 μm were demonstrated. It should be noted that the drug delivery as well as other task execution capabilities of our Ebots is of high repeatability. In fact, in addition to the control of a single Ebot to execute such tasks, multiple Ebots can be controlled simultaneously to execute the biological tasks by controlling the light intensity and illumination duration. As shown in Fig. S22, three drug-loaded Ebots were simultaneously navigated toward three targeted cells in a cell cluster for target drug delivery and selective cell killing.

In addition to the targeted drug delivery, the Ebot also can be used for the selective removal of diseased cells in cell clusters. As shown in Fig. 4d, at *t* = 8 s, diseased cells were effectively removed, and the morphology and position of the remaining cells were unaffected. This targeted drug delivery capability is very important for intestinal drug delivery and further biomedical applications. To demonstrate this ability, as shown in Fig. 4e, DLSP-loaded Ebot can be navigated among the gaps (with a width of only 5 μm) of adipocytes in the gut. After effective drug release, the Ebot was re-used to remove the mesoporous silica particle from the gap without affecting the gut (Fig. 4f).

Most algae have photosynthesis functions, and this photosynthesis capability can be explored for further use. For example, Roxby et al. reported that microalgae can be turned into a living optical resonator for enhanced biophotocurrent generation based on the photosynthesis function^51^. In our case, our EG-based Ebot also has this photosynthesis function. EG is rich in photosynthetic pigments such as chlorophyll, so it can absorb light for photosynthesis and convert light energy into chemical energy (adenosine triphosphate: ATP) and produce oxygen for further use. During the process of light capturing/absorption for photosynthesis, chlorophyll is at excited state after light absorption, it acts as a reaction center and plays an important role in electron transfer at the electron transfer chain on the thylakoid membrane. During this process, the excited electrons are transferred through excited chlorophyll to a series of membrane carriers, such as pheophytin and quinones, and finally for the synthesis of ATP and coenzyme nicotinamide adenine dinucleotide phosphate (NADPH).

**Fig. 5:**
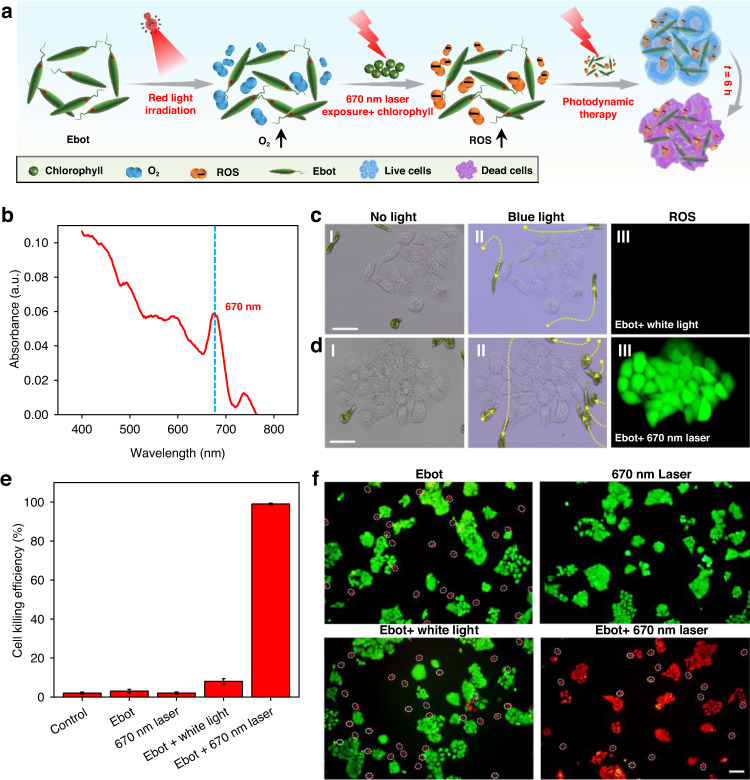
Ebot for photodynamic therapy (PDT). **a** Schematic illustration of PDT using Ebot. **b** Light absorption of EG. **c**, **d** Experimental results showing Ebot navigation and ROS generation. (I, II) Microscopic images showing navigation of Ebot to designated locations with blue light irradiation, (III) Fluorescent images showing the generation of ROS (green fluorescence) with different light irradiation. **e** Histogram showing the efficiency of PDT under different treatments of Ebot. **f** Fluorescent images showing the results of PDT with different treatments of Ebot. HeLa cells stained with Calcein-AM (green, live cells) and PI (red, dead cells), the white dashed circle represents Ebots. Scale bar: 50 μm. Data for (**e**) are presented as mean values ± s.d. (*n* = 16)

In addition to the conventional photosynthesis capability, our Ebot can be further used for PDT at targeted locations (Fig. 5a). Due to the strong absorption of chlorophyll at 670 nm (Fig. 5b), the photosynthesis efficiency is much higher when the Ebot is irradiated with red light than that with white light. O_2_ is produced due to the photosynthesis process. Importantly, chlorophyll can be reduced to chlorophyll derivatives, such as chlorins, under red light irradiation. As a natural photosensitizer, chlorins can be excited from a ground state to an excited state under the irradiation of 670 nm laser beam, which can then convert the produced O_2_ into reactive oxygen species (ROS)^52^. This feature makes our Ebot a natural candidate for PDT which can significantly improve the hypoxic environment of tumor. In the experiments, Ebots were first navigated to the targeted location of HeLa cells via blue light irradiation (Fig. 5c, d). Then 670-nm laser beam (50 mW) was irradiated on the cells with a duration of 10 min, the cells were then incubated for 6 h. As shown in Fig. 5d, ROS was observed with ROS fluorescence probes (2’, 7’-dichlorodihydrofluorescein diacetate (DCFH-DA), green). However, such ROS signal was not observed with white light irradiation (Fig. 5c). After the observation of ROS generation capability of our Ebot, we further show that the ROS can be directly used for cancer cell killing via PDT. As shown in Fig. 5e and f, effective PDT was realized with the treatment of our Ebot and 670-nm light irradiation, and up to 99% Hela cells were killed (Propidium iodide-PI, red fluorescence), while with other treatments, most of the cells were still alive (Calcein-AM, green fluorescence). The results in Fig. 5 demonstrate that our Ebot has the potential for PDT.

## Discussion

In summary, we developed a light-controlled multifunctional soft bio-microrobot (Ebot) based on *Euglena gracilis* (EG) that can execute different biomedical tasks such as targeted drug delivery and precision removal of diseased cells in narrow microenvironments, as well as PDT. By irradiation with blue light with an intensity of 3000 lx, the Ebot shows controllable motion and navigation capability. In addition, the Ebot also shows controllable deformation by the light stimulation, allowing it to pass through different narrow and complicated microfluidic channels. These controllable motion and deformation capabilities allow the Ebot to execute different biomedical tasks such as targeted drug delivery and selective removal of diseased cells in cell clusters, without affecting neighboring cells. The targeted drug delivery and removal capability is also demonstrated in the intestinal tract in vitro. Importantly, the application of PDT in targeted location is also demonstrated using our Ebot.

Although different biohybrid microrobots have also been reported by integrating living cells (for example sperm, bacteria) or cell membrane with synthetic materials (mostly magnetic materials)^53–55^, additional magnetic materials and large magnetic devices are needed to navigate their movements. In addition, most of the biohybrid microrobots do not have the ability of deformation, which limits their applications in complex and narrow environments. Compared with these conventional biohybrid microrobots, there are three main advantages of our Ebot: First, no magnetic materials or other synthetic materials are needed to modify the microrobot, a simple LED can realize controllable navigation. Second, our Ebot also has flexible deformation ability, so the Ebot can be used in complex and narrow biological microenvironments. Finally, due to the photosynthesis of EG and its rich content of chlorophyll, our Ebot can be used as a natural PDT tool. For further applications in vivo, one of the major challenges is the precise delivery of light and manual guidance of Ebot. Due to the limitation of light penetration depth as well as light scattering in the in-vivo environment, it will face challenges for the effective delivery of light to control the Ebot in vivo. Fortunately, the emerging of biomedical optical fibers that can be used for effective light delivery in vivo with high biocompatibility may provide a new solution for the problem of current in-vivo light delivery. In addition, for the in vivo applications, there would be an immune rejection problem due to the foreign biomaterials. For this challenge, there are two possible solutions. First, we can use the mammalian neutrophil membrane for Ebot coating to reduce immune rejection. Due to the uniqueness of the neutrophil membrane, it can effectively reduce immune rejection. Another solution to reduce the immune response is to encapsulate Ebot into degradable oral capsules. By combining the phototactic movement of Ebot with the protective ability of oral capsules, the Ebot can be navigated to a targeted position in the digestive system for further use. When the Ebot is navigated to the lesion site in the digestive system, the Ebot will be released from the capsule. After task execution, the Ebot are eventually degraded and broken down into biological fragments due to the high biodegradable ability. This light-controlled soft bio-microrobot serves as a versatile bio-microrobotic tool, which holds great potentials for biomedical task execution in different narrow and complicated positions where conventional tools are difficult to access due to the lack of deformability and bio-adaptability.

## Materials and methods

### Culture of EG

EG and HUT medium were purchased from the Freshwater Algae Culture Collection at the Institute of Hydrobiology, FACHB, Wuhan, China. First, EG cells were cultured at room temperature (25 °C) after shaking in a test tube. Second, the sample was illuminated in a light incubator with a 12/12-hour day-night cycle by light-emitting diodes (450 nm, 100 lx). Finally, the sample was resuspended in EG culture medium for further experiments.

### Preparation of positively charged DLSP

DOX was purchased from Melone Pharmaceutical Co., Ltd, Shanghai, China. Mesoporous silica particles (MSPs) with a diameter of 1 μm were purchased from Huake Microtechnology Co., Ltd, Wuhan, China. Lysine was purchased from Aladdin Biochemical Technology Co., Ltd, Shanghai, China. FITC was purchased from Yeasen Biotechnology Co., Ltd, Shanghai, China. To load DOX, 50 mg of DOX was dissolved in 5 mL deionized water (DI) at a mass concentration of 10 mg/mL. Then, 100 mg MSP was added to 5 mL DOX solution. It was stirred for 24 hours at 25 °C in a shaker at 180 rpm. Finally, it was centrifuged at 2000 rpm for 10 min, and the supernatant was removed to obtain a DLSP solution. Lysine was diluted tenfold, and 5 mL diluted lysine was added to the DLSP sample. The sample was stirred for 6 h on a magnetic stirrer. Positively charged 1-μm mesoporous silica particles loaded with DOX were obtained. The DLSP pellets were placed in a 1 mL centrifuge tube. At the same time, FITC with a mass concentration of 1 mg/mL was added to the centrifuge tube. The mixture was incubated at room temperature for 4 h in the dark. Finally, a green fluorescent DLSP was obtained.

### Fabrication of microfluidic channels

Microfluidic mazes and channels were designed with AutoCAD. Design drawings were processed by Dx fluidics Co., Ltd. Jiangsu, China. Silicone elastomer and curing agent were mixed in a mass ratio of 10:1. The mixture was placed in a vacuum pump for 2 hours to remove air bubbles during the mixing process. Finally, bubble-free polydimethylsiloxane (PDMS) was obtained. Bubble-free PDMS was poured on the SU8 photoresist mold and cured in an oven at 70 °C for 3 hours. Then, the PDMS was peeled off from the mold. Finally, microfluidic channels were obtained.

### Cultured cells

Fetal Bovine Sera (FBS), Penicillin-Streptomycin Solution (P/S), and DMEM were mixed to obtain a complete medium. HeLa cells were cultured in Petri dishes containing a complete medium, and then slides were placed in the dishes. Next, the dishes were placed in an incubator at 37 °C with 5% CO_2_ for 48 h until the cells on the slide reached 70%-80% confluence.

### Cell Viability Test

The cell viability assay kit (calcein-AM) was purchased from KeyGEN BioTECH Corp., LTD, Jiangsu, China. First, the complete medium in the cells was removed, then 2 mL of PBS was added. 2 μL calcein-AM staining solution was added to the cells, and then were placed in a dark environment and incubated for 30 min. Next, cells were washed twice with PBS buffer.

### Preparation of In vitro Intestines

Fresh pig intestines were purchased from local markets, and the pig intestines were washed multiple times with Phosphate-Buffered Saline (PBS) solution to remove intestinal contents. Then the soft intestinal organ was cut into pieces (3 × 3 mm) using medical scissors. After the preparation of the intestine tissue, Ebot solution were injected onto the intestine through a disposable sterile syringe for drug delivery experiments. For the imaging/light delivery during the drug delivery process, we focused on a region of microscope field of view (length: 630 μm, width: 450 μm) under a ×20 objective.

### Supplementary information

Supplementary Information

Movie S1

Movie S2

Movie S3

Movie S4

Movie S5

Movie S6

Movie S7

Movie S8

## Data Availability

All data are available in the main text or the supplementary materials.
