# Supplementary material for: Light-controlled soft bio-microrobot
Source: Light Sci Appl. 2024 Feb 26;13:55. doi: 10.1038/s41377-024-01405-5 (PMC10894875; doi:10.1038/s41377-024-01405-5)
Supplement: Supplementary file 1 — Supplementary Information [file 41377_2024_1405_MOESM1_ESM.docx]

**Supplementary Information for**

**Light-controlled soft bio-microrobot**

Jianyun Xiong^1#^, Xing Li^1#^, Ziyi He^1^, Yang Shi^1^, Ting Pan^1^, Guoshuai Zhu^1^, Dengyun Lu^1^, and Hongbao Xin^1*^

^1^ Guangdong Provincial Key Laboratory of Nanophotonic Manipulation, Institute of Nanophotonics, Jinan University, Guangzhou 511443, China

^#^ J. X. and X. L. contributed equally to this work.

* To whom correspondence may be addressed

**Email:** [hongbaoxin@jnu.edu.cn](mailto:hongbaoxin@jnu.edu.cn)

**This Word file includes:**

Fig. S1. EG characterization.

Fig. S2. EG trajectory.

Fig. S3. Ebot motion control under illumination of different light.

Fig. S4. Schematic illustration of movement and deformation mechanism of EG.

Fig. S5. Control of rotation motion of Ebot.

Fig. S6. Ebot trajectory without light irradiation.

Fig. S7. Simultaneous control of two Ebots under three motion modes.

Fig. S8. Light control of CR and *volvox*.

Fig. S9. The degree of deformation of the Ebot under different light intensities.

Fig. S10. Without light source, the passing through a 2D straight microfluidic

channel of Ebot.

Fig. S11. Failure of passing through microfluidic channel without light irradiation.

Fig. S12. Failure of passing through a curved channel without light irradiation.

Fig. S13. Co-culturing of EG with HeLa cells for 1 day.

Fig. S14. Biodegradation process of EG in SIF.

Fig. S15. EG Cell viability as a function of time for different treatments.

Fig. S16. DOX loading on mesoporous silica particles.

Fig. S17. Ebot modification with DLSP.

Fig. S18. Ebot swimming in different biological media.

Fig. S19 Influence of pH and temperature on the motion speed of Ebot.

Fig. S20. Moving and delivery of 200 nm PS particles and particle cluster using Ebot.

Fig. S21. Loading and release of particles with different sizes by Ebot.

Fig. S22. Simultaneously navigation of three drug-loaded Ebots for targeted drug delivery and selective cell killing.

**Other Supplementary Materials for this manuscript include the following:**

Movie S1. Three locomotion patterns of Ebot at different light intensities.

Movie S2. Polygonal movement of Ebot at medium light intensity.

Movie S3. Different turning angles of Ebot at high light intensity.

Movie S4. Comparison of Ebot’s traversal of a micro-maze with and without light.

Movie S5. Deformation progress of Ebot.

Movie S6. Comparison of Ebot’s passing through the 2D microchannels with and without light irradiation.

Movie S7. Comparison of Ebot’s passing through the 3D microchannels with and without light irradiation.

Movie S8. Comparison of Ebot’s passing through the curved microchannels with and without light irradiation.**
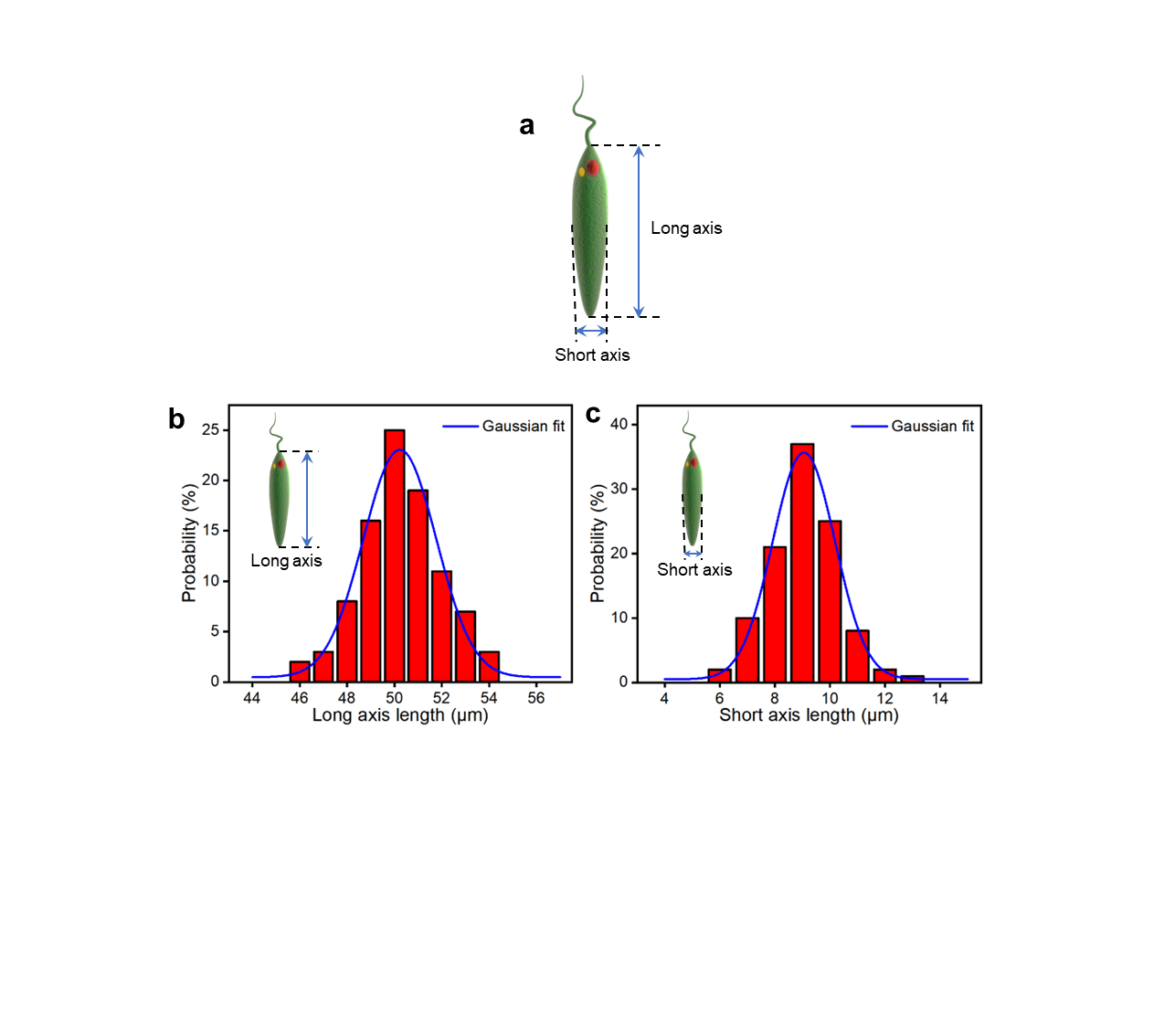
**

**Fig. S1. Size characterization of EG cells.** (**a**) Schematic illustration of a EG cell. (**b**) Histogram of the length of the long axis with Gaussian fit, the peak value of the length is 50 µm. (**c**) Histogram of the length of the short axis with Gaussian fit, the peak value of the length is 9 µm.


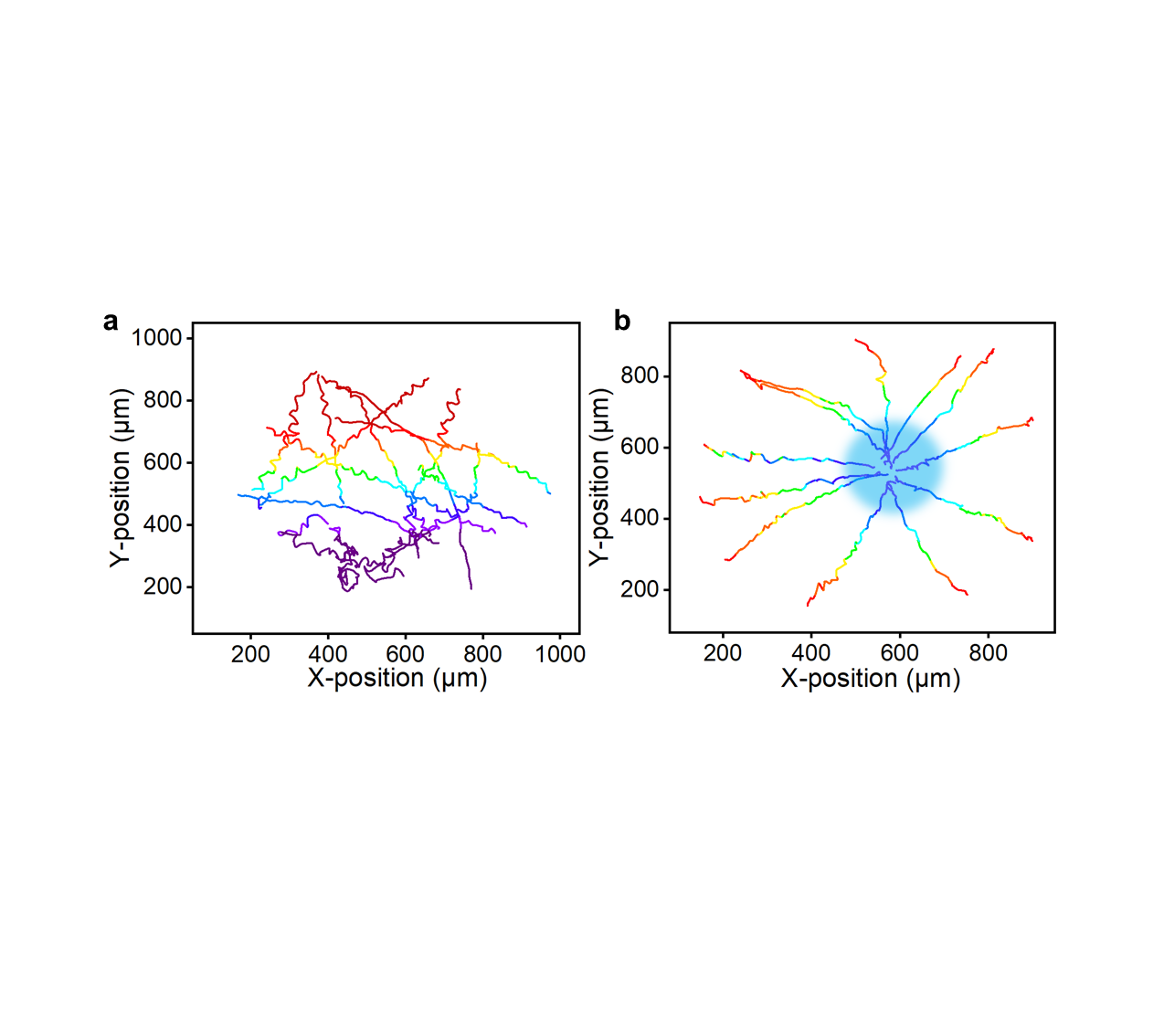


**Fig. S2.** Trajectory of the EG during (**a**) natural swimming and (**b**) phototatic swimming.


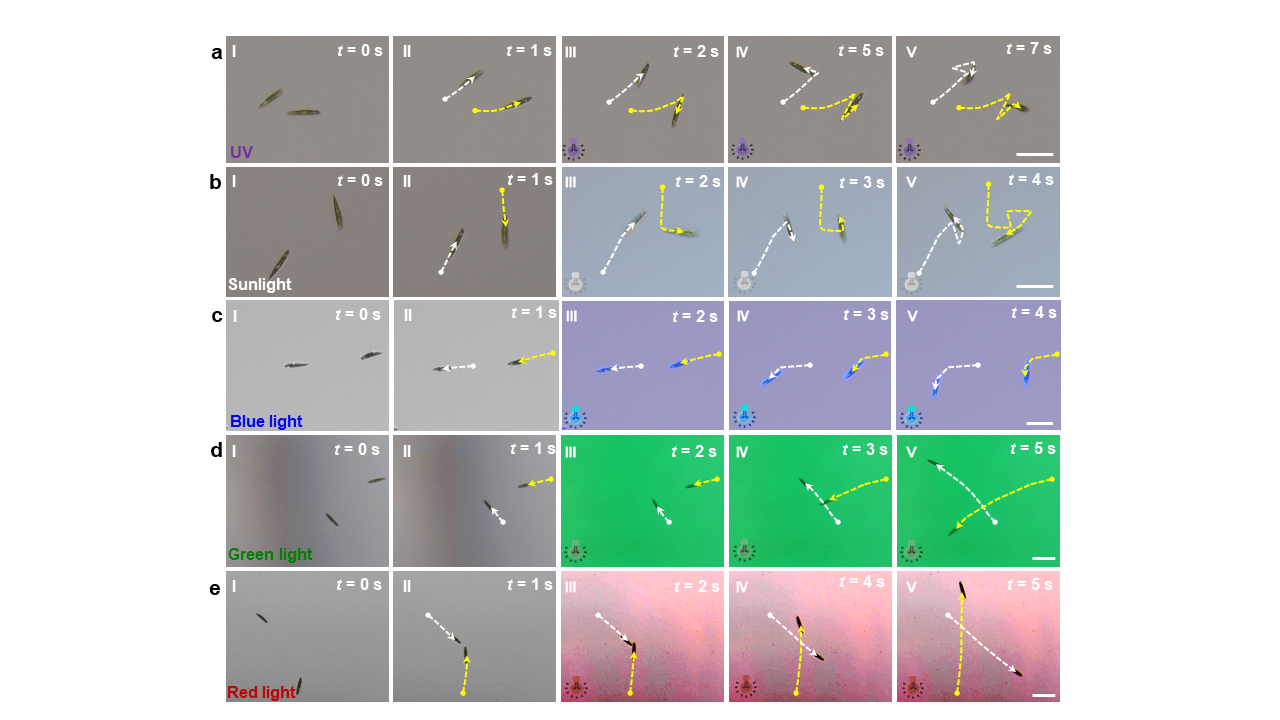


**Fig. S3.** Ebot motion control under illumination of different light of (**a**) UV, (**b**) sunlight, (**c**) blue light, (**d**) green light, (**e**) red light. Light is on at *t* = 2 s, the motion of two Ebots are effectively and synchronously changed with blue light irradiation. Scale bars: 50 μm.


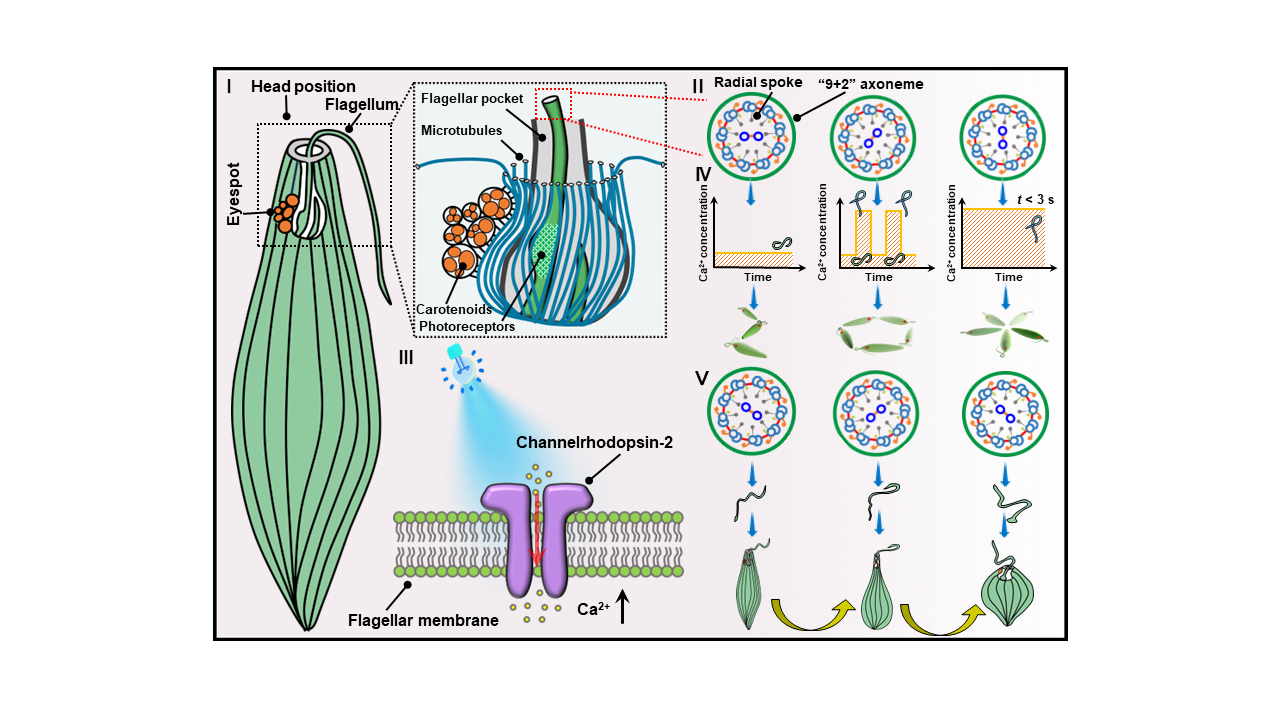


**Fig. S4.** Schematic illustration of EG movement and deformation mechanism.


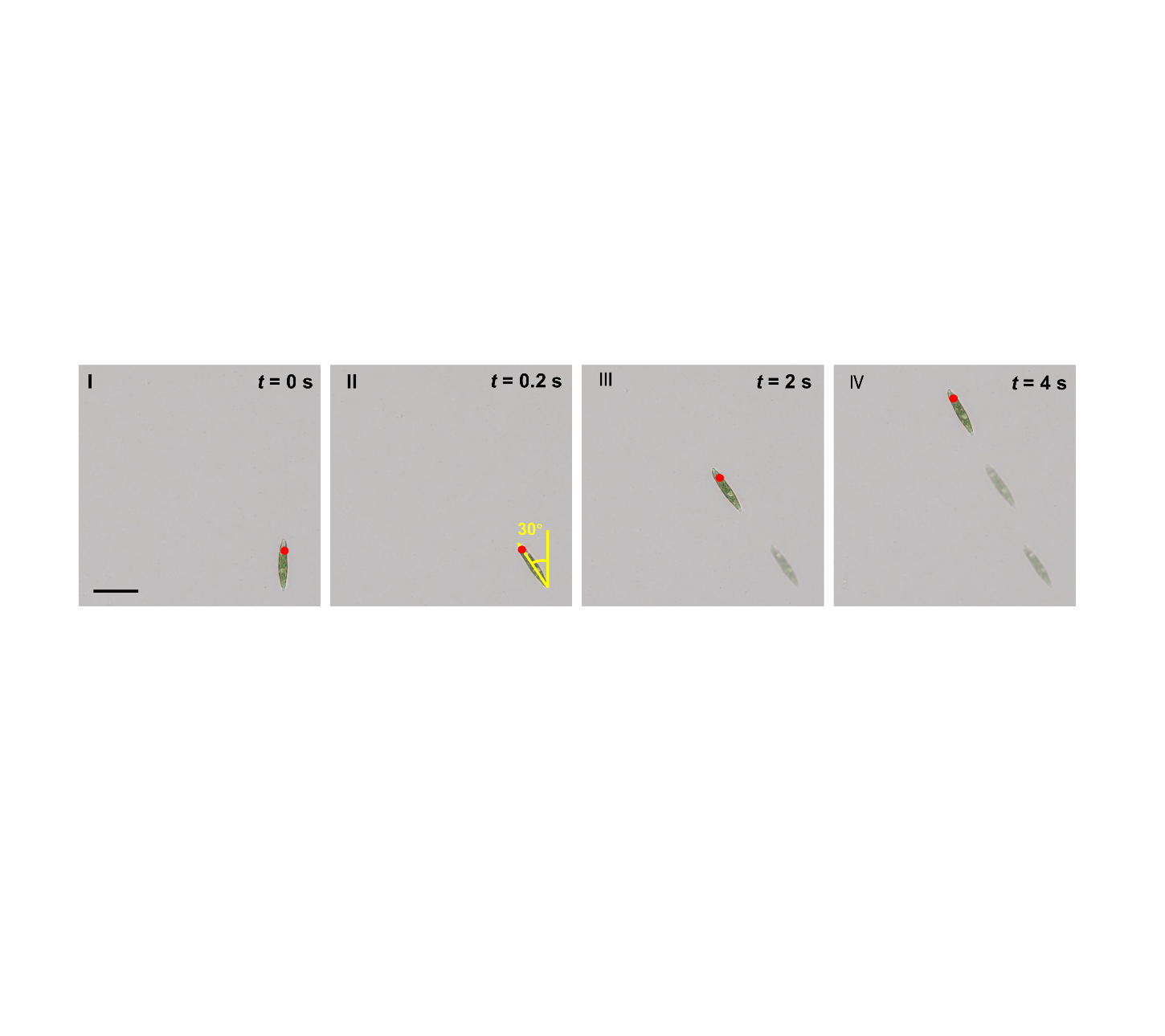


**Fig. S5.** **Control of rotation motion of Ebot.** Panel I: original moving direction. Panel II: under 3000 lx irradiation, Ebot was controlled to rotate with a specific angle of 30° at a given light duration. Panel III and IV: after turning off the light, Ebot swims helically. Scale bar: 20 μm.


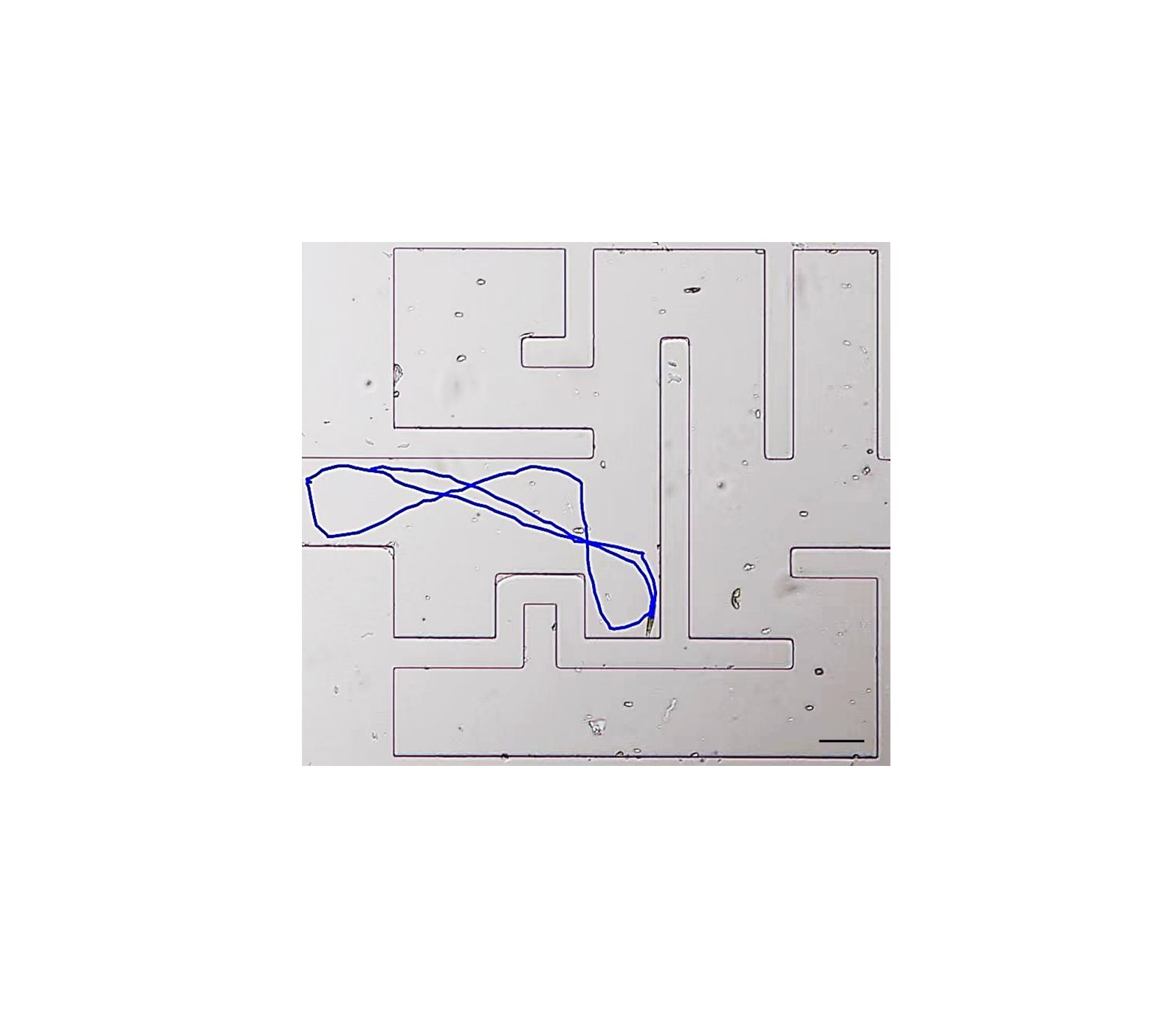


**Fig. S6.** Microscopic image shows that without light control, Ebot was lost in microfluidic maze, blue curve indicates the Ebot trajectory. Scale bar: 50 μm.

**
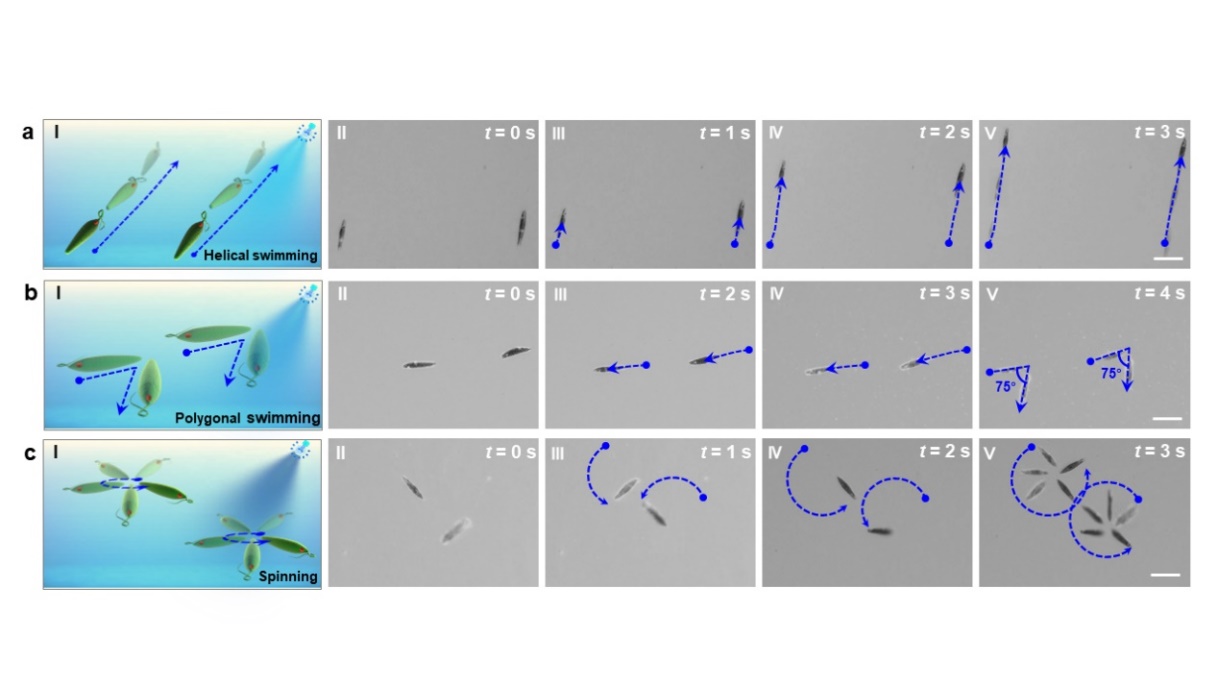
**

**Fig. S7. Simultaneous control of two Ebots under three motion modes** (**a**) helical swimming under low light intensity (100 lx), (**b**) polygonal swimming under medium light intensity (1000 lx), and (**c**) spinning swimming under high light intensity (3000 lx). Panels I: schematic illustration of these three motion modes, blue curves show the motion strategy. Panels Ⅱ-Ⅳ: microscopic images showing the movement of two Ebots. Panels Ⅴ: microscopic images show final swimming trajectories with several images superimposed (blue curves indicated). Scale bars: 50 μm.


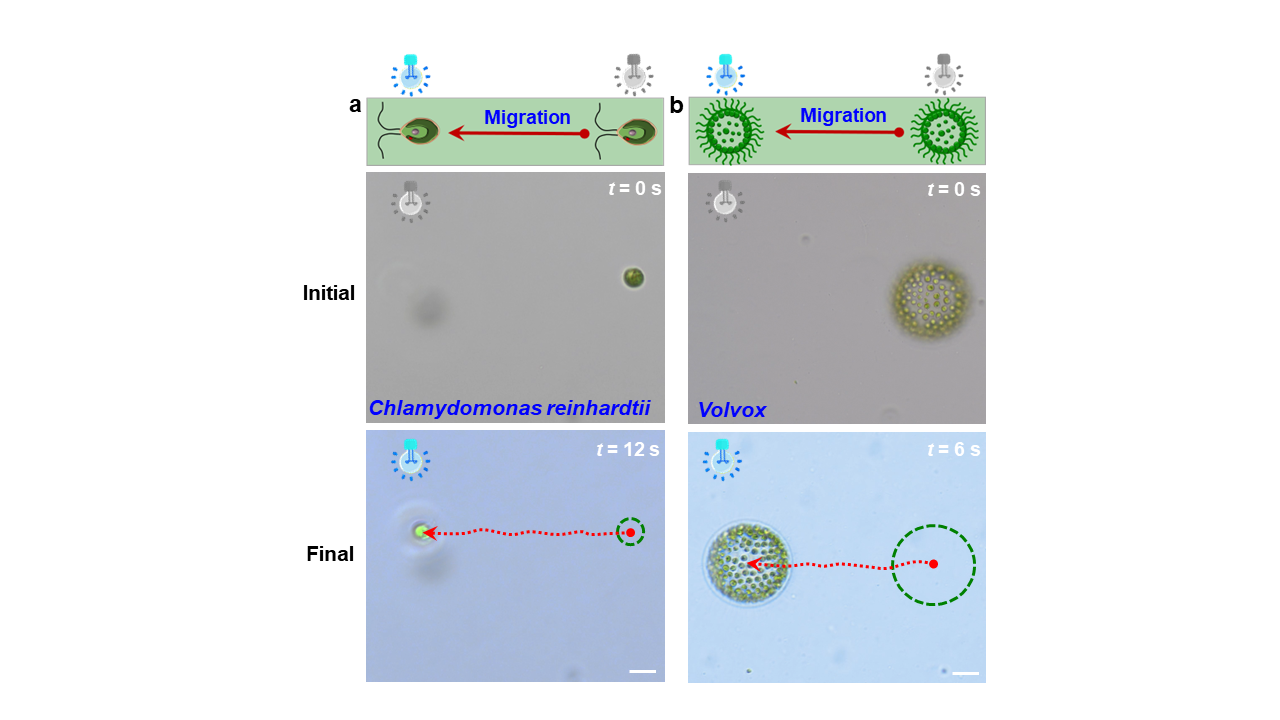


**Fig. S8.** **Light control of (a) CR and (b) *volvox*.** The red dotted line indicates the movement trajectory, and the green dotted circle indicates the origin position of CR and *volvox*. Scale bars: 10 μm.


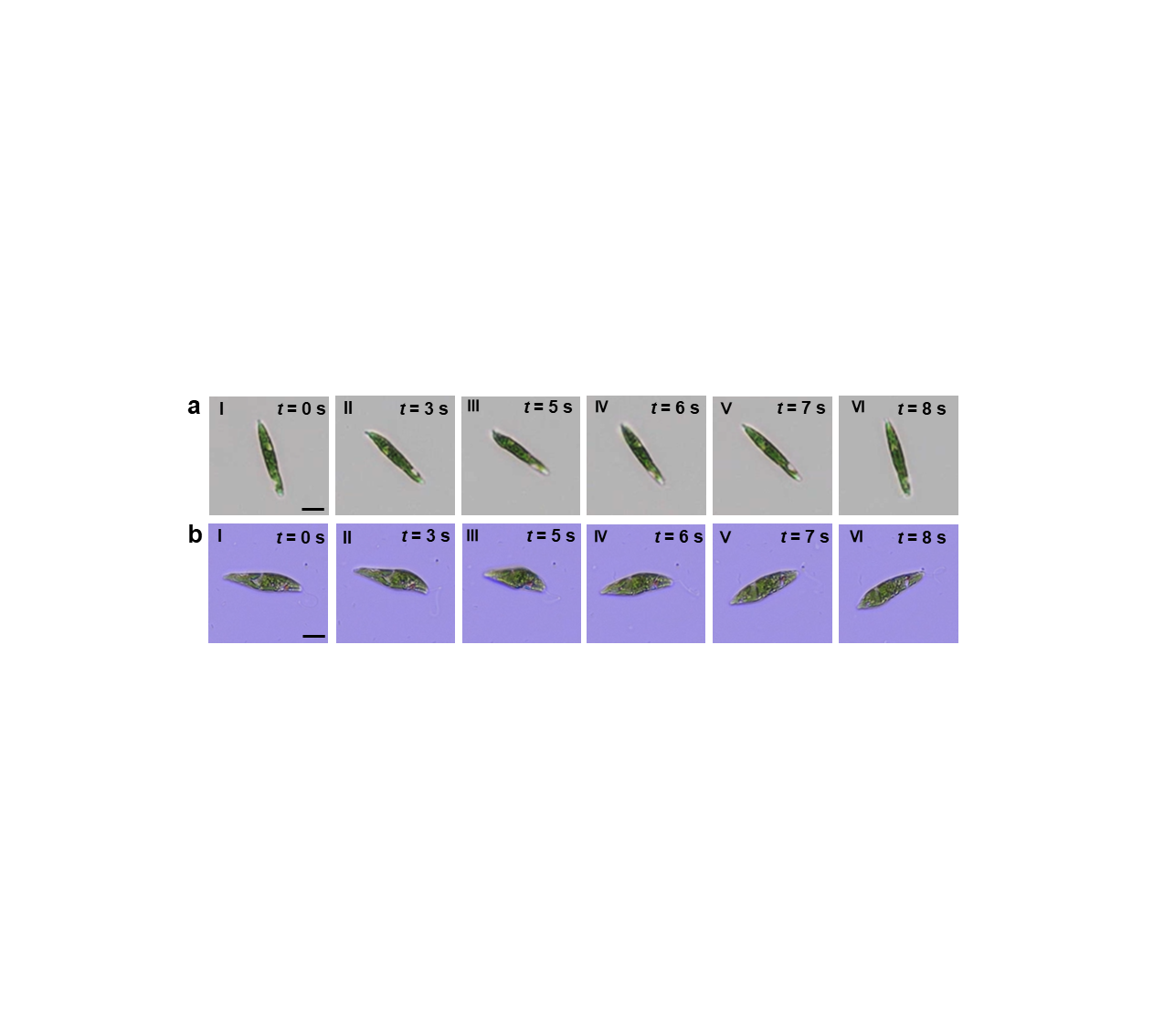


**Fig. S9.** **The degree of deformation of the Ebot under different light intensities.** (**a**) Low light intensity (100 lx). (**b**) Medium light intensity (1000 lx). Scale bar: 10 μm.


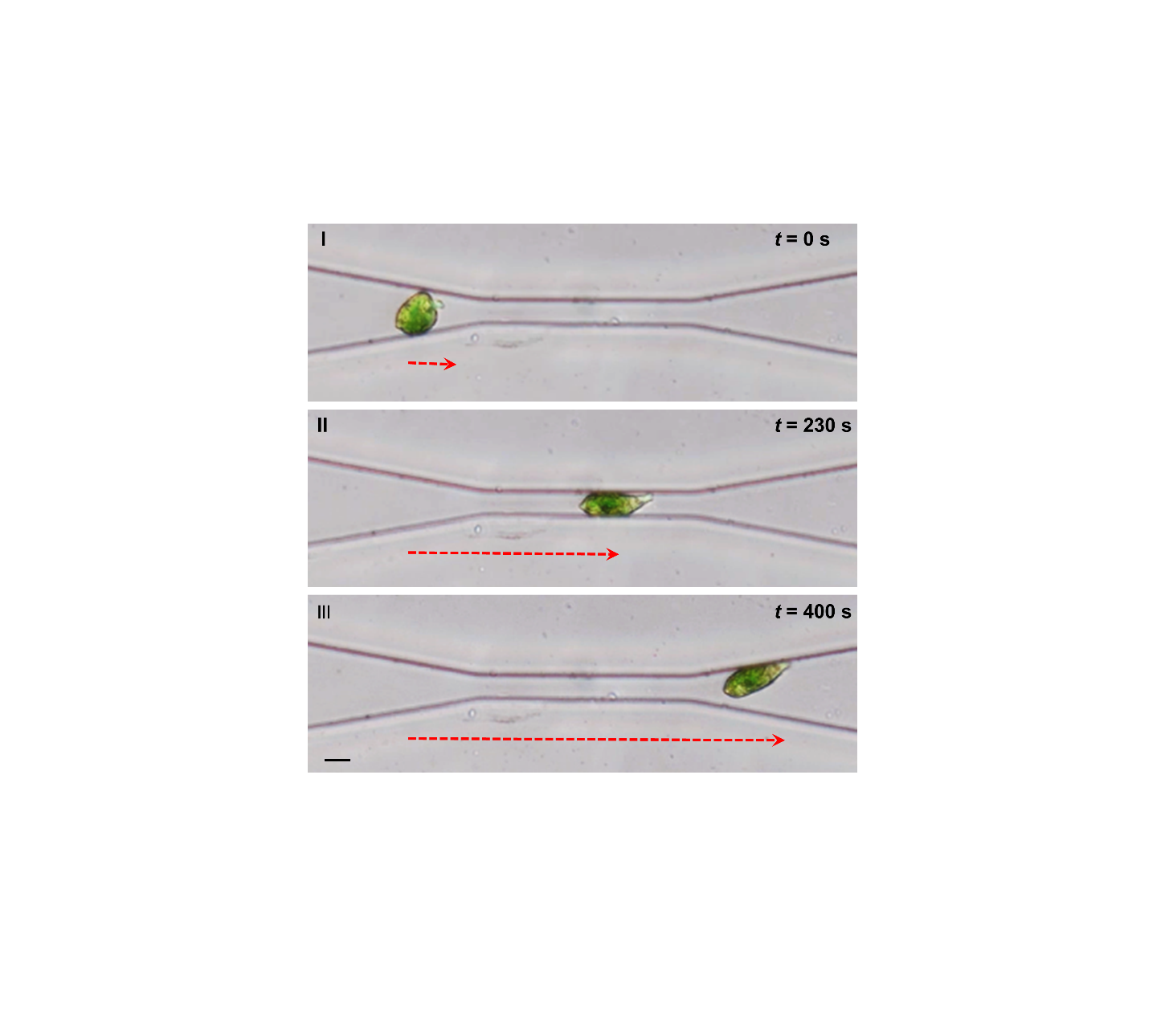


**Fig. S10.** Without light source, the Ebot pass through a 2D straight microfluidic channel (width: 10 μm). The red dashed line indicates the movement direction of the Ebot. Scale bar: 10 μm.


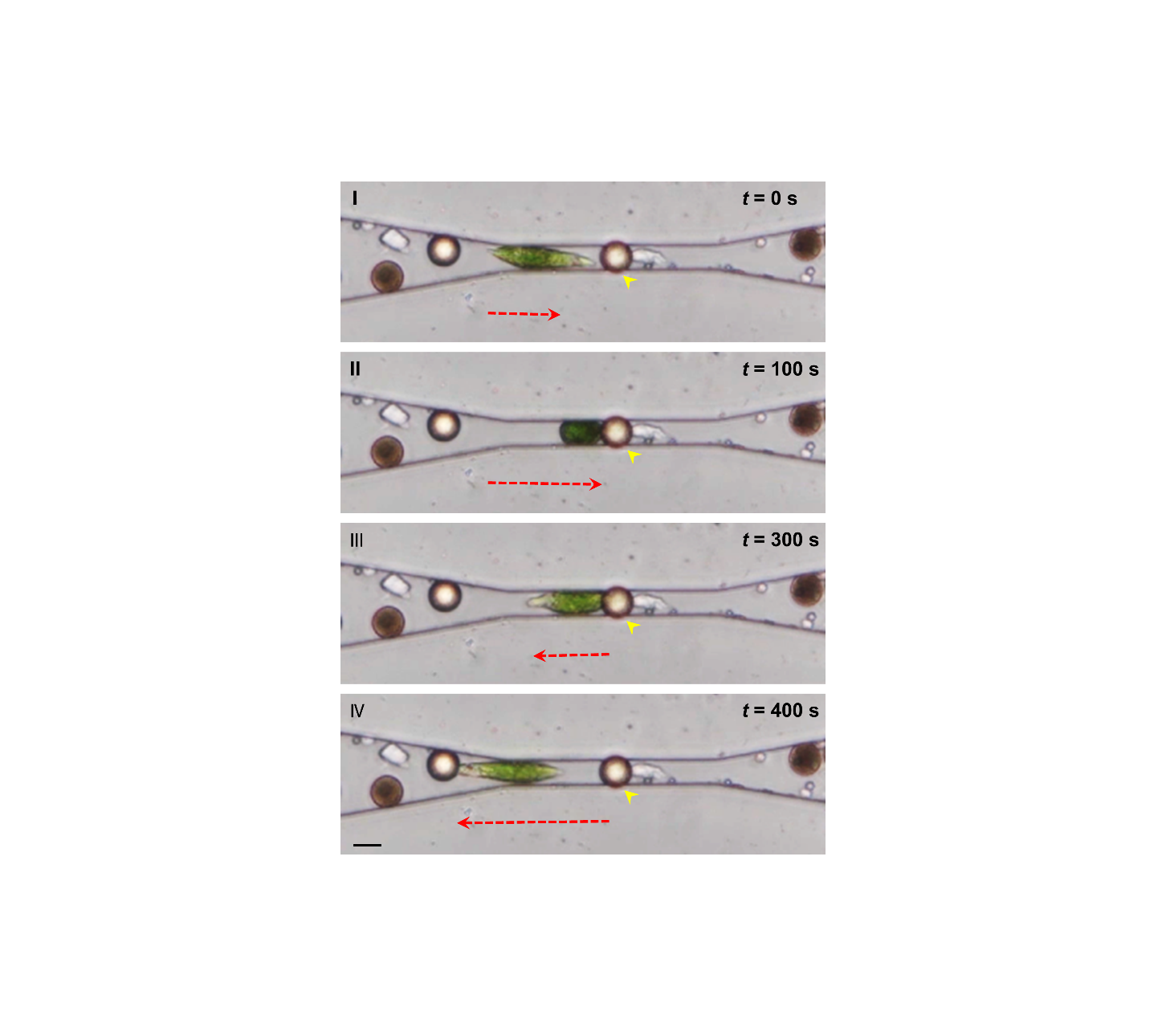


**Fig. S11.** **Failure of passing through microfluidic channel without light irradiation.** (Ⅰ, Ⅱ) Encountering an obstacle. (Ⅲ, Ⅳ) Turning back. The red dashed line indicates the movement direction of the Ebot. The yellow arrows indicate obstacles. Scale bar: 10 μm.


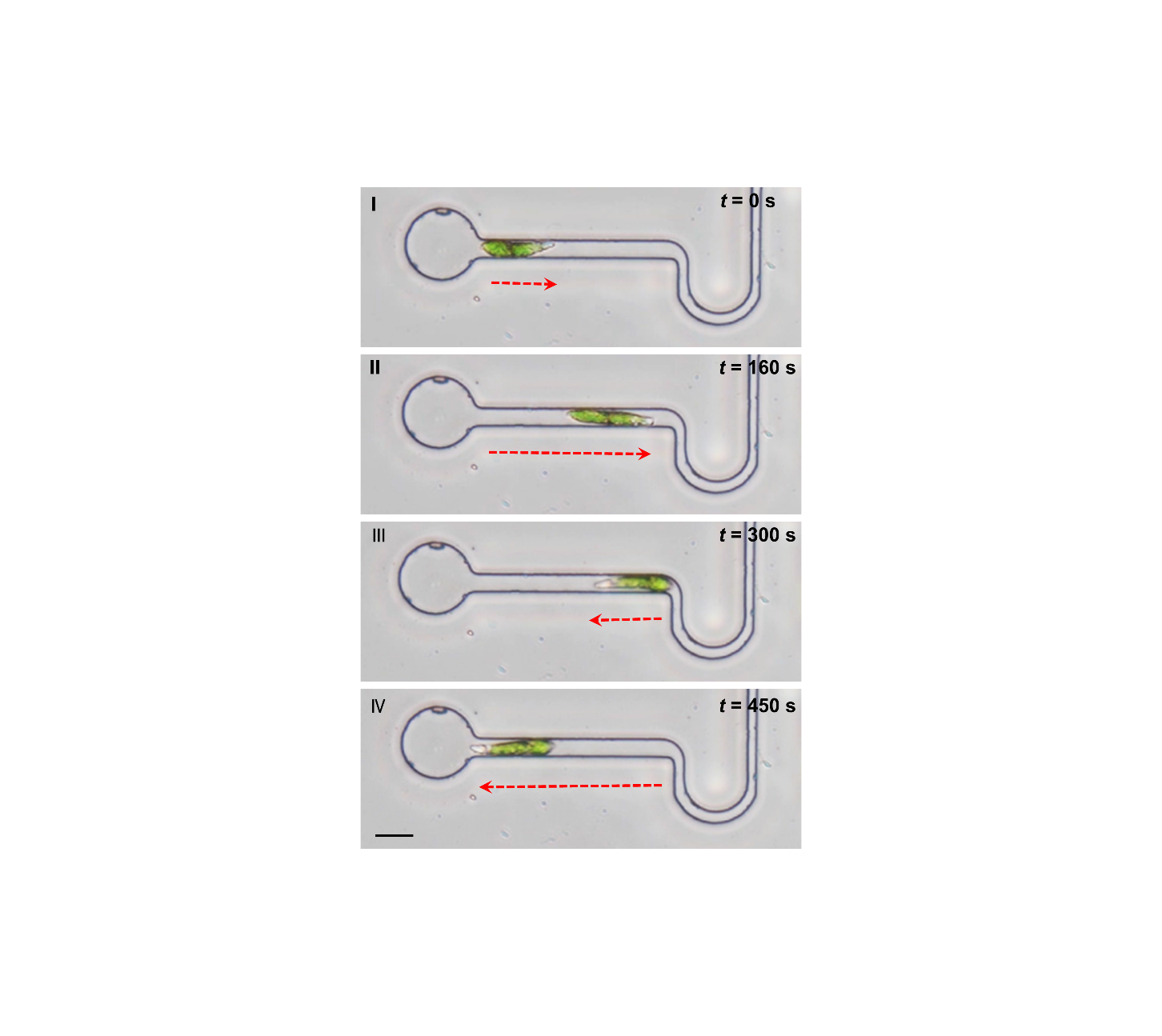


**Fig. S12.** **Failure of passing through a curved channel without light irradiation.** (I, II) Moving toward the curved part of the channel. (Ⅲ, Ⅳ) Turning back after approaching the turning point. The red dashed line indicates the moving direction of the Ebot. Scale bar: 10 μm.


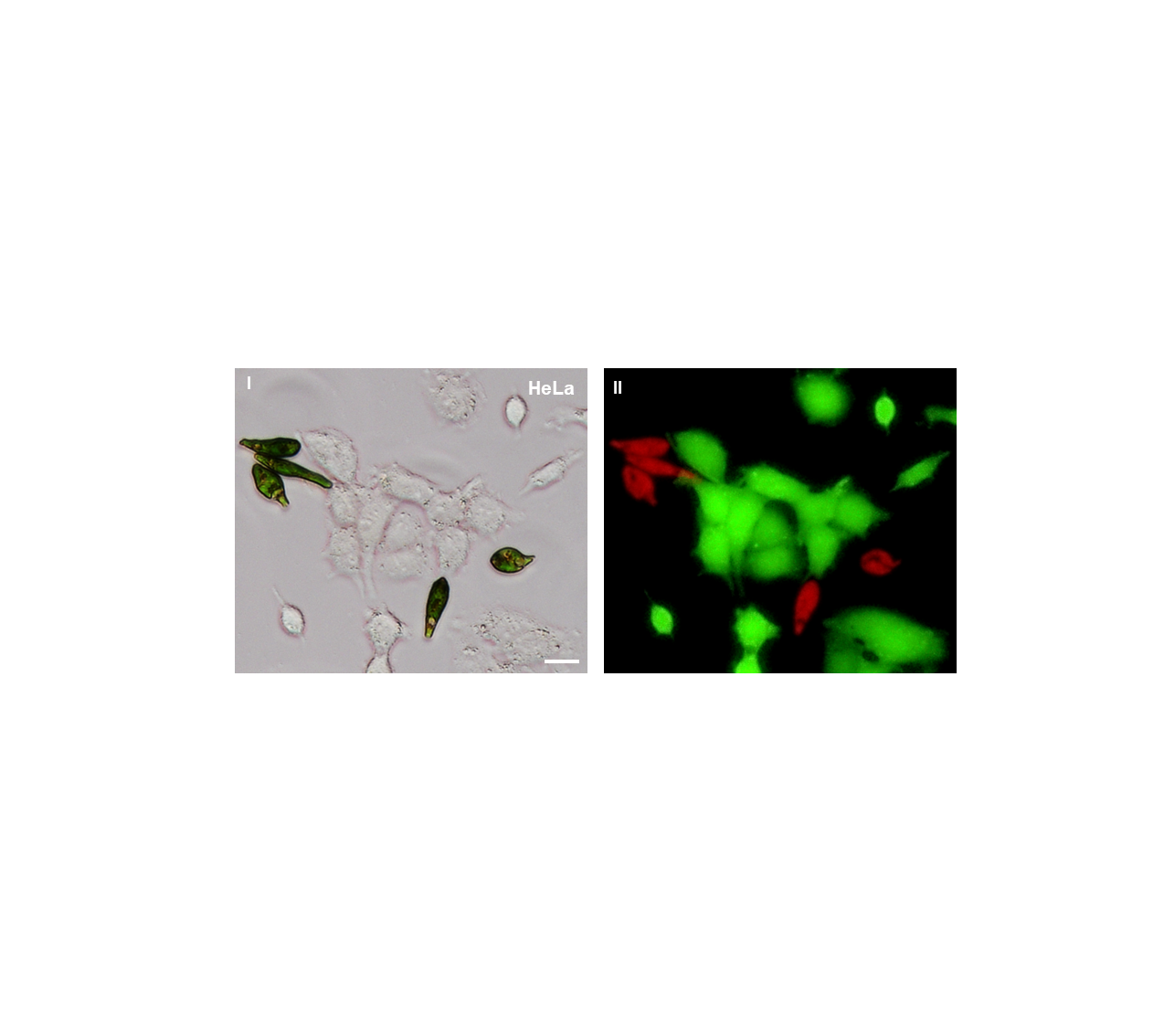


**Fig. S13. Co-culturing of EG with HeLa cells for 1 day.** (Ⅰ) Bright-field image showing the EG with HeLa cells. (Ⅱ) Merged image showing the EG and HeLa cells. Green fluorescence for live cells, red fluorescence for EG. Scale bar: 20 μm.

**
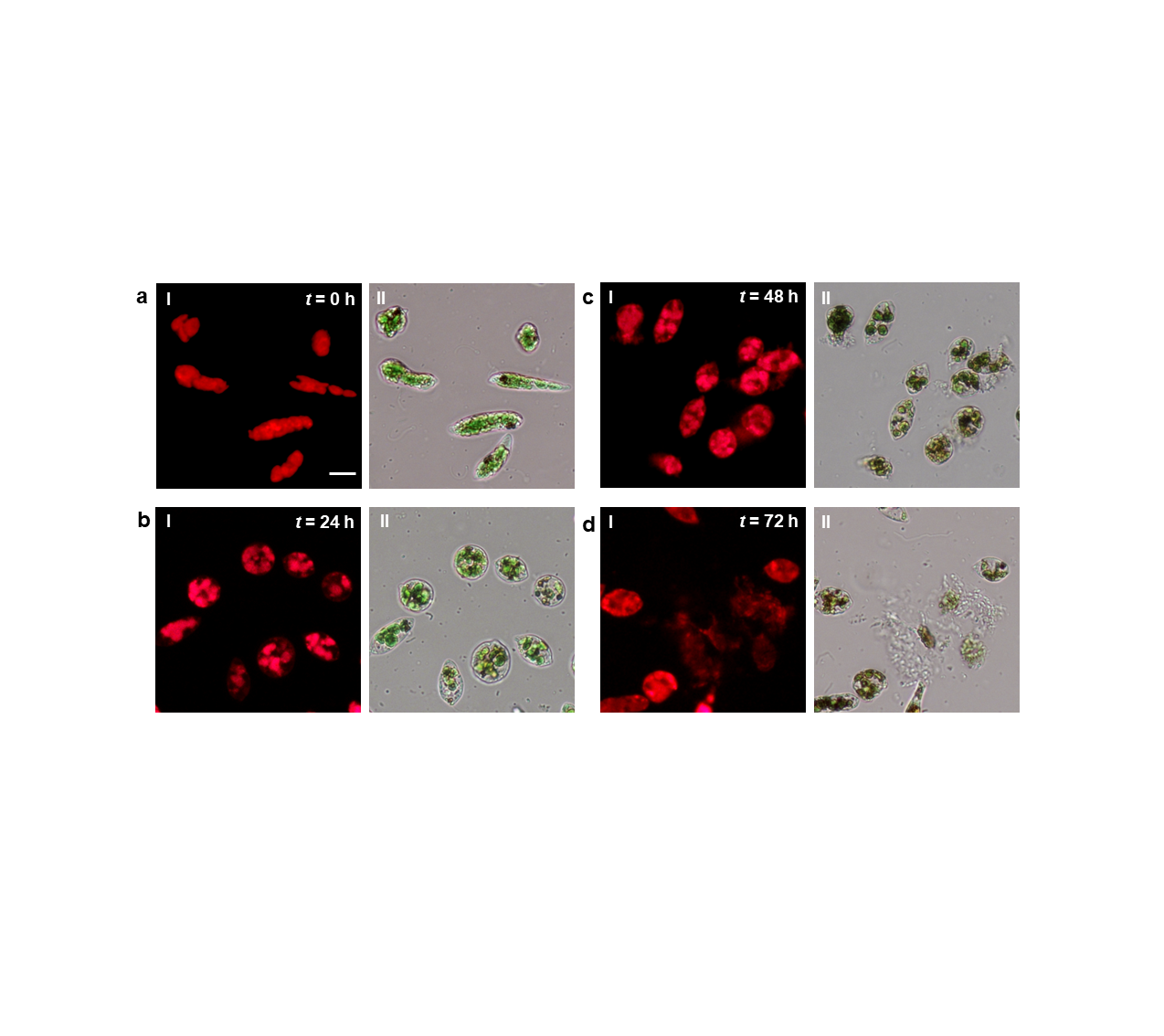
**

**Fig. S14. Biodegradation process of EG in SIF.** (**a**) Initial state. (**b-d**) after culturing of (**b**) 24 hours, (**c**) 48 hours, and (**d**) 72 hours, (Ⅰ) fluorescence images (Ⅱ) corresponding bright field images. Scale bar: 10 μm.


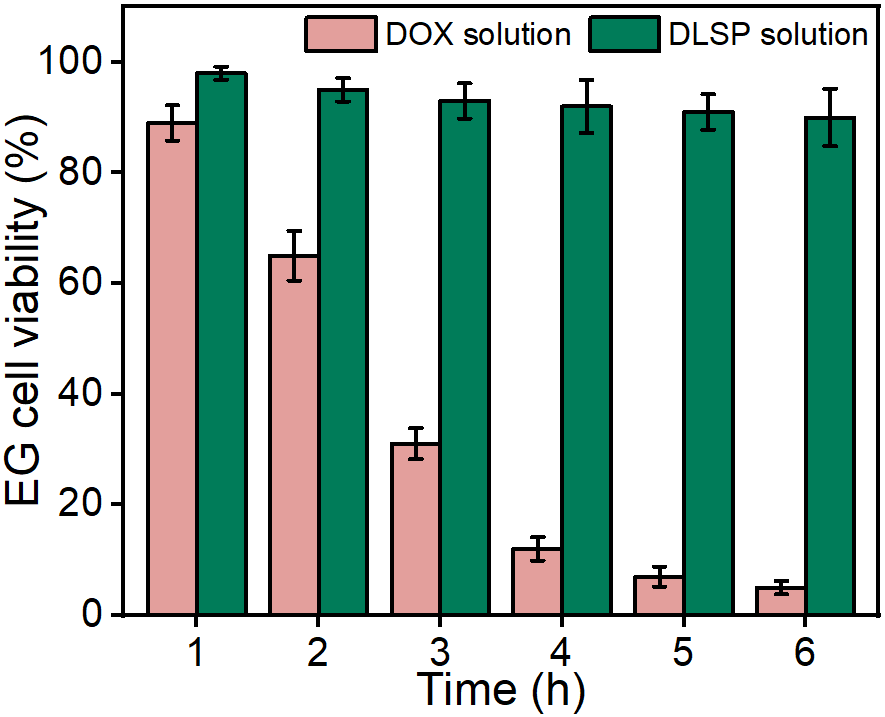


**Fig. S15. EG Cell viability as a function of time for different treatments.**


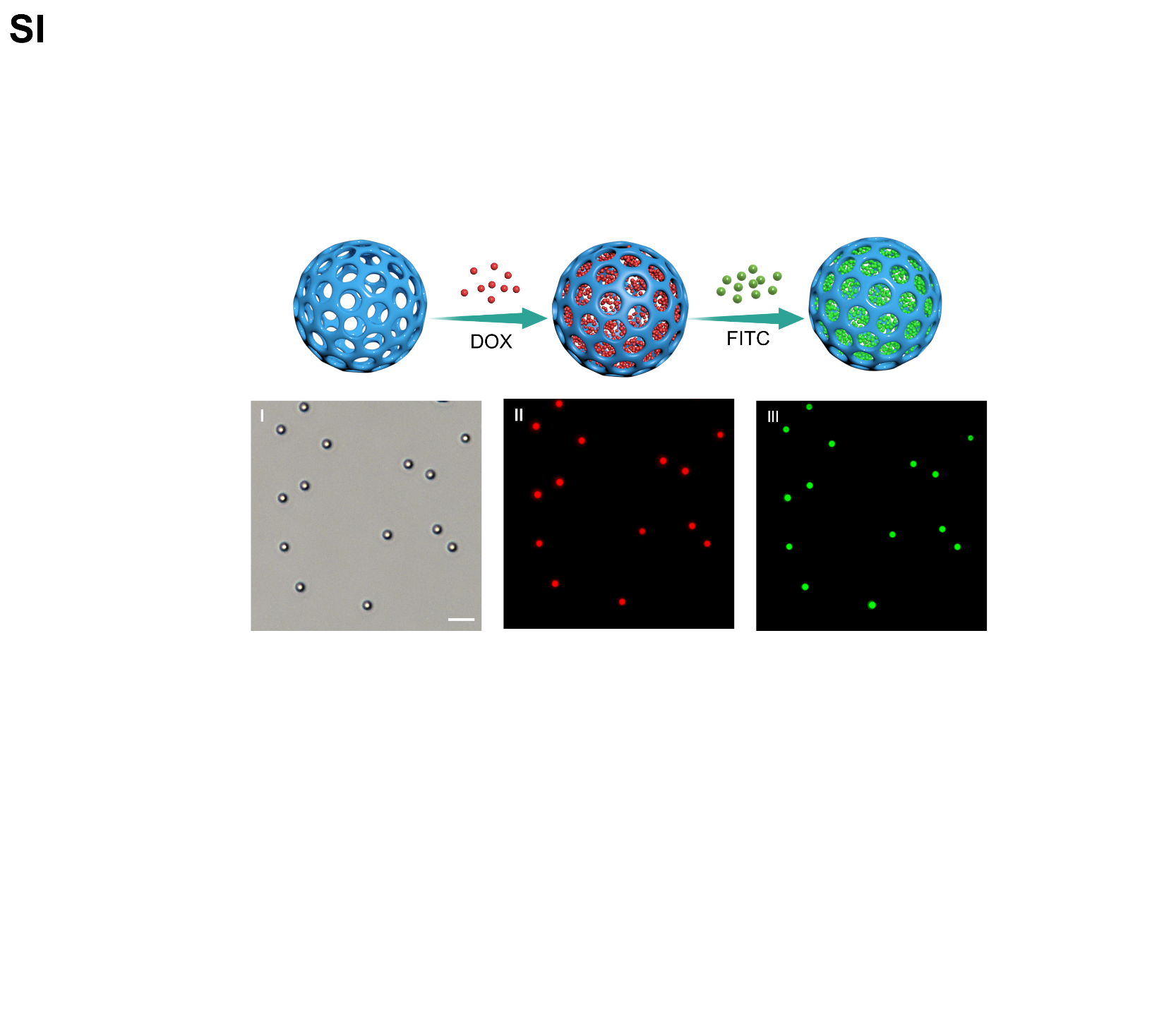


**Fig. S16.** **DOX loading on mesoporous silica particles.** Upper: Schematic illustration. Lower: (I) bright field image, (II) fluorescence image showing particle loaded with DOX (red), (III) fluorescence image showing particle loaded with FITC (green). Scale bar: 5 μm.

**
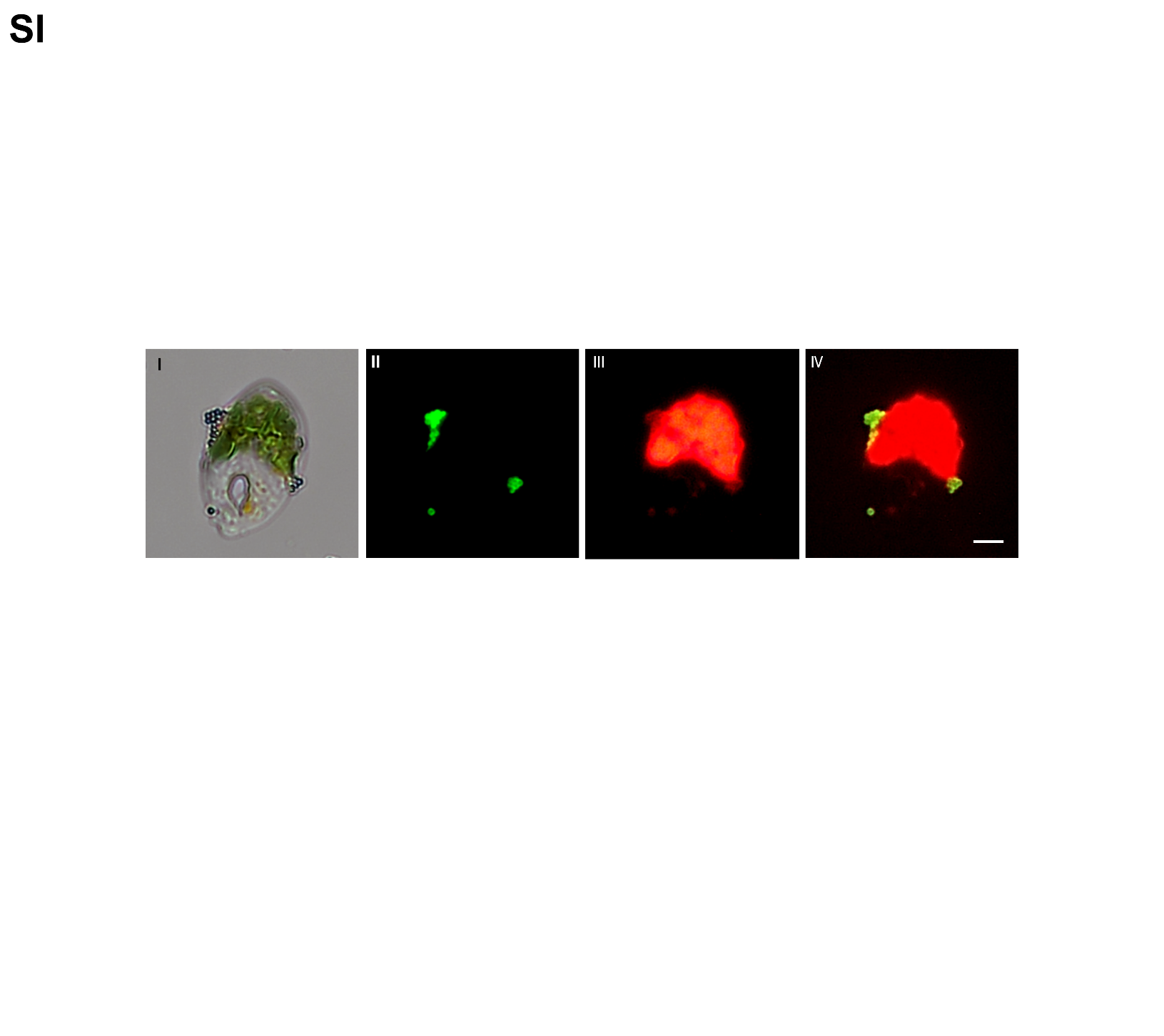
**

**Fig. S17.** **Ebot modification with DLSP.** (Ⅰ) Bright field image. (Ⅱ) Fluorescence image (green fluorescence for DLSP modified with FITC). (Ⅲ) Fluorescence image (red fluorescence for Ebot and DLSP). (Ⅳ) Merged image. Scale bar: 3 μm.

**
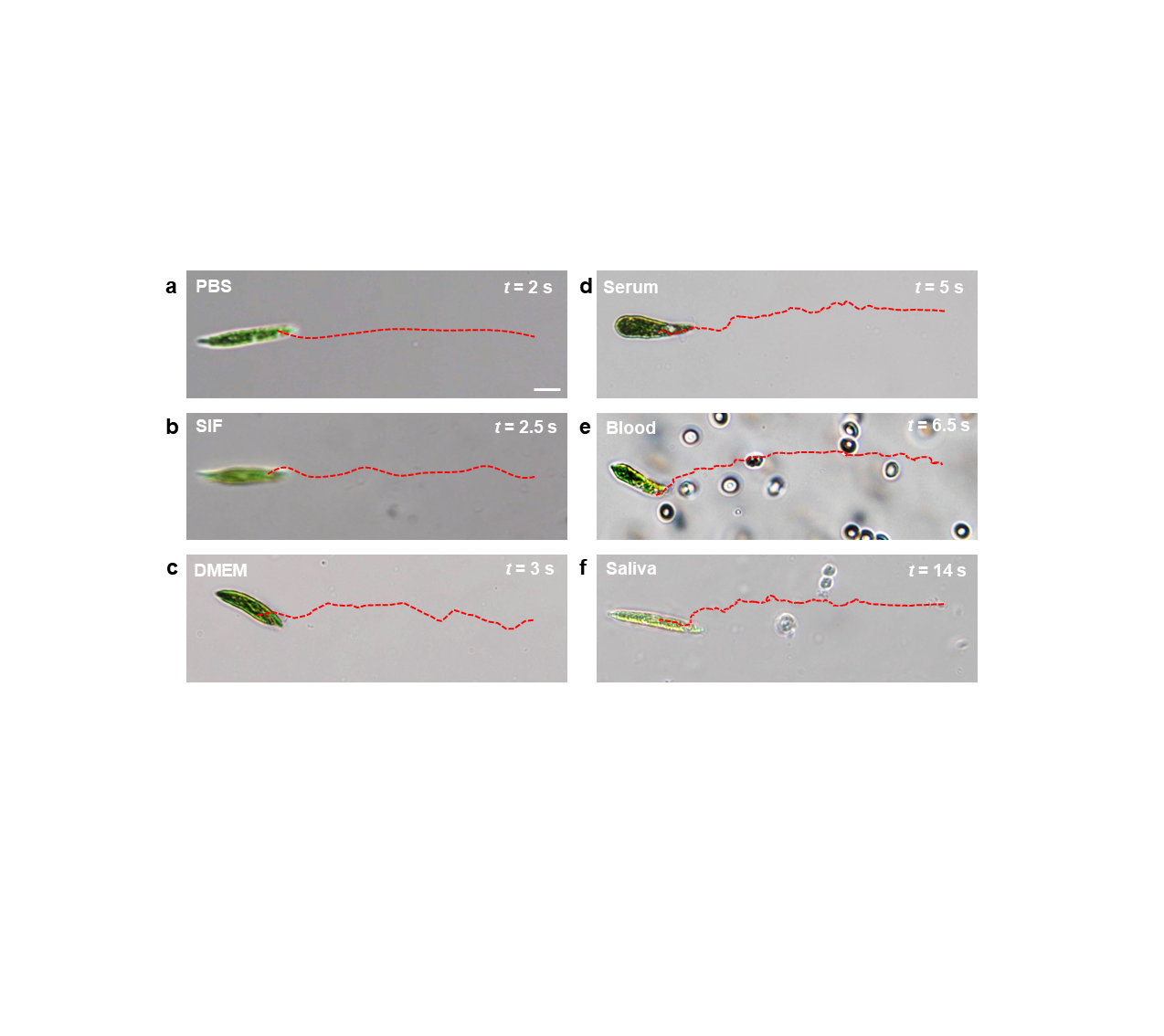
**

**Fig. S18.** **Ebot swimming in different biological media.** (**a**) PBS. (**b**) SIF. (**c**) DMEM. (**d**) Serum. (**e**) Blood. (**f**) Saliva. Scale bar: 10 μm.

The movement speed of Ebot in a neutral (pH = 7) and weakly alkaline (pH = 8) environment was similar to that in the normal culture media. However, the movement performance was greatly reduced in acid or strong alkali environments. While for the temperature, the Ebot can maintain a high speed (about 60 μm/s) at the temperature of 25 °C. Both the decrease and increase in temperature can reduce the moving speed.

**
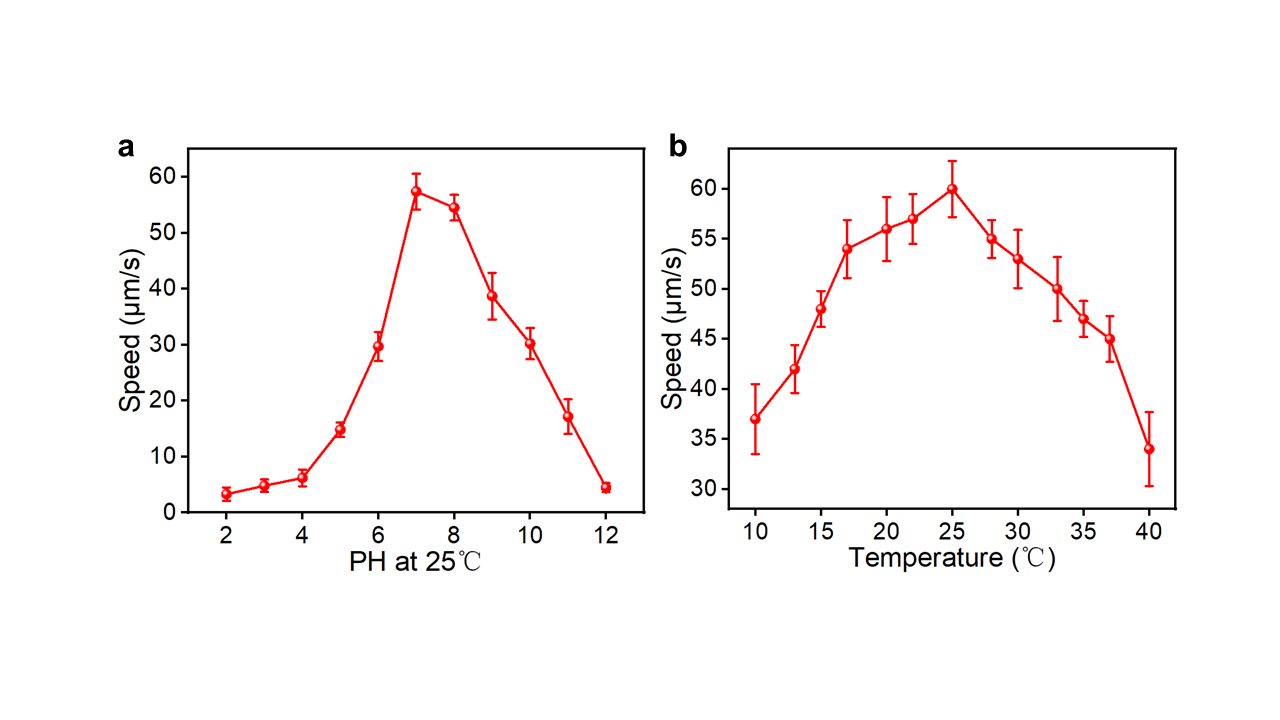
**

**Fig. S19** Influence of (**a**) pH and (**b**) temperature on the motion speed of Ebot.


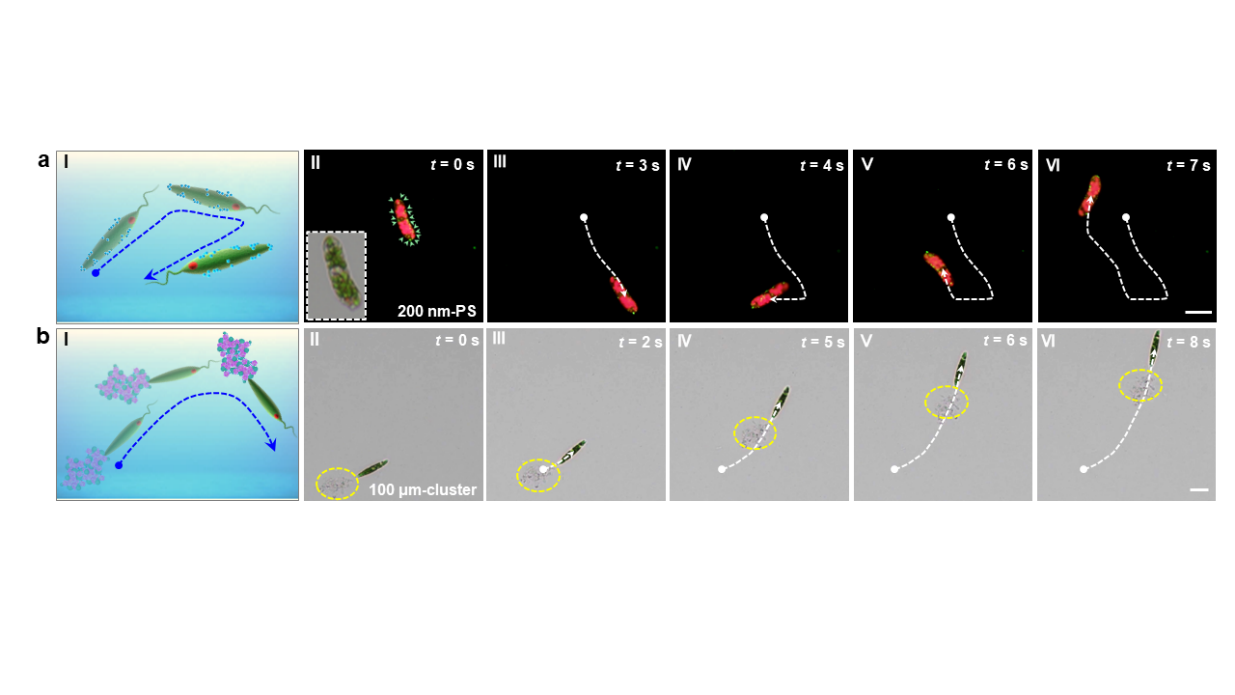


**Fig. S20. Moving and delivery of (a) 200 nm PS particles and (b) particle cluster using Ebot.** Panels Ⅰ are schematic of nanoparticle/cluster moving and delivery by Ebot. (**a**) Ebot was loaded with 200 nm fluorescent PS particles. Inset in panel II shows a brightfield image. Panels Ⅱ-Ⅵ are fluorescence images (Ebot is red fluorescent, 200 nm particles indicated by green arrows are green fluorescent). White curve shows the moving trajectory. (**b**) Ebot dragging 100 μm particle cluser (yellow circle indicated). Panels Ⅱ-Ⅵ are brightfield images. Scale bars: 30 μm.


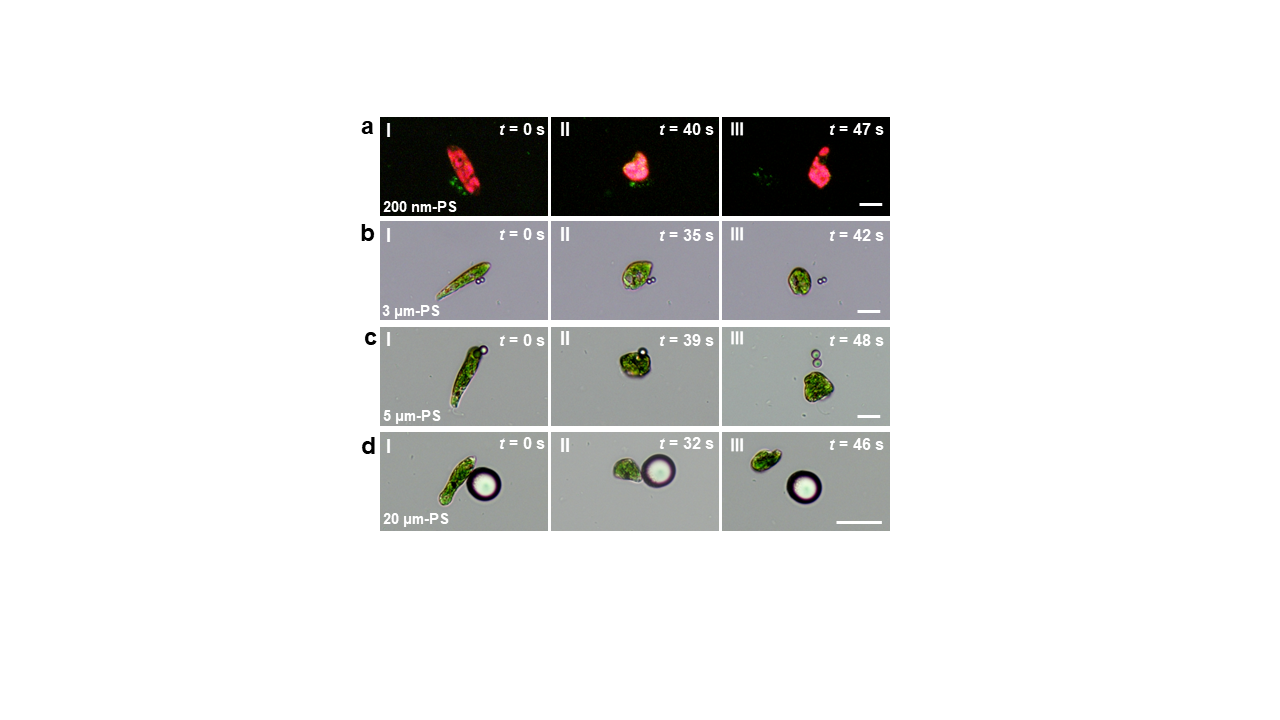


**Fig. S21. Loading and release of particles with different sizes by Ebot.** (**a**) Fluorescence image showing loading and release of 200 nm PS particles. (**b-d**) Bright field images showing loading and release of PS particles with sizes of (**b**) 3 μm (**c**) 5 μm (**d**) 20 μm. Scale bars: 30 μm.

**
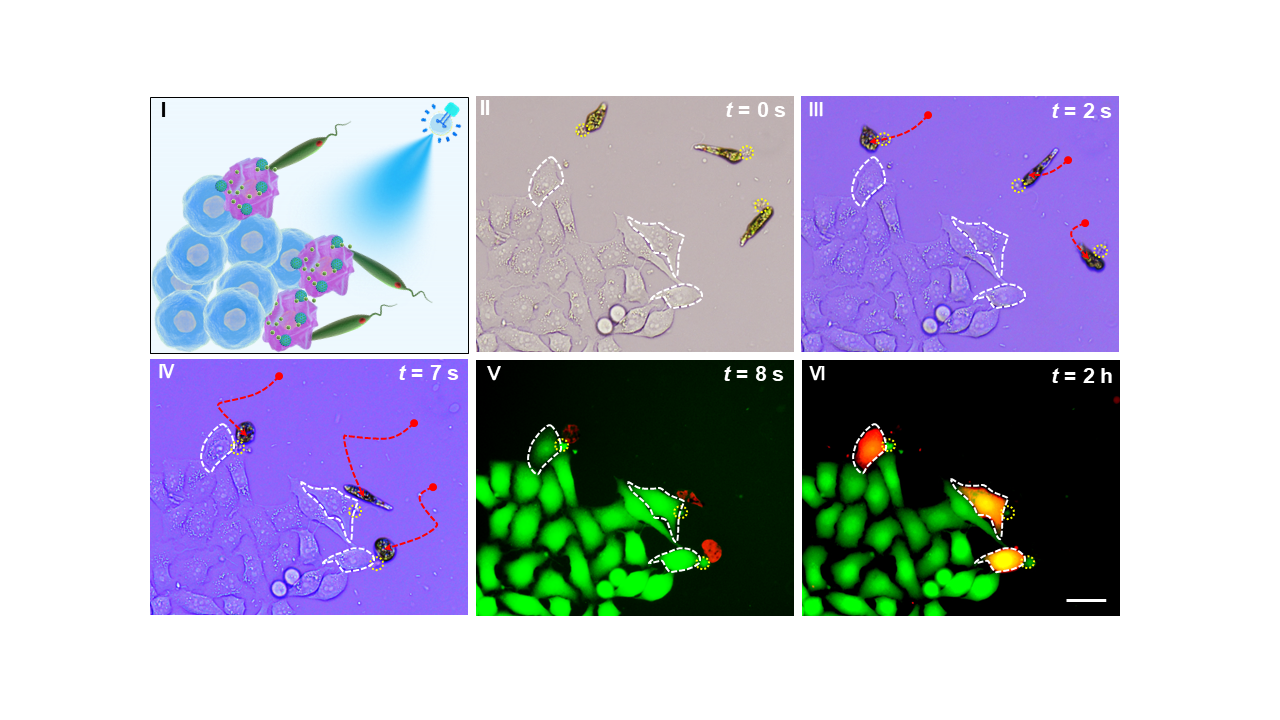
**

**Fig. S22. Simultaneously navigation of three drug-loaded Ebots for targeted drug delivery and selective cell killing.** Panel Ⅰ: a schematic diagram showing this performance, Panels Ⅱ-IV: simultaneously targeted drug delivery process of three drug-loaded Ebots, Panels Ⅴ-Ⅵ: fluorescent images showing drug release and selective killing of Hela cells, live HeLa cells are in green fluorescent. The yellow and white dashed circle indicates the DLSP and cultured HeLa cells, respectively. Red dashed curves show the trajectory of the Ebot. Scale bars: 30 μm.
